# Cooperativity of Oncogenic K-Ras and Downregulated p16/INK4A in Human Pancreatic Tumorigenesis

**DOI:** 10.1371/journal.pone.0101452

**Published:** 2014-07-16

**Authors:** Zhe Chang, Huaiqiang Ju, Jianhua Ling, Zhuonan Zhuang, Zhongkui Li, Huamin Wang, Jason B. Fleming, James W. Freeman, Dihua Yu, Peng Huang, Paul J. Chiao

**Affiliations:** 1 Department of Molecular and Cellular Oncology, The University of Texas MD Anderson Cancer Center, Houston, Texas, United States of America; 2 Sun Yat-sen University Cancer Center, State Key Laboratory of Oncology in South China, Collaborative Innovation Center for Cancer Medicine, Guangzhou, China; 3 Department of Pathology, The University of Texas MD Anderson Cancer Center, Houston, Texas, United States of America; 4 Department of Surgical Oncology, The University of Texas MD Anderson Cancer Center, Houston, Texas, United States of America; 5 The Division of Hematology and Medical Oncology, Department of Medicine, The University of Texas Health Science Center at San Antonio, San Antonio, Texas, United States of America; H.Lee Moffitt Cancer Center & Research Institute, United States of America

## Abstract

Activation of K-ras and inactivation of p16 are the most frequently identified genetic alterations in human pancreatic epithelial adenocarcinoma (PDAC). Mouse models engineered with mutant K-ras and deleted p16 recapitulate key pathological features of PDAC. However, a human cell culture transformation model that recapitulates the human pancreatic molecular carcinogenesis is lacking. In this study, we investigated the role of p16 in hTERT-immortalized human pancreatic epithelial nestin-expressing (HPNE) cells expressing mutant K-ras (K-ras^G12V^). We found that expression of p16 was induced by oncogenic K-ras in these HPNE cells and that silencing of this induced p16 expression resulted in tumorigenic transformation and development of metastatic PDAC in an orthotopic xenograft mouse model. Our results revealed that PI3K/Akt, ERK1/2 pathways and TGFα signaling were activated by K-ras and involved in the malignant transformation of human pancreatic cells. Also, p38/MAPK pathway was involved in p16 up-regulation. Thus, our findings establish an experimental cell-based model for dissecting signaling pathways in the development of human PDAC. This model provides an important tool for studying the molecular basis of PDAC development and gaining insight into signaling mechanisms and potential new therapeutic targets for altered oncogenic signaling pathways in PDAC.

## Introduction

Pancreatic ductal adenocarcinoma (PDAC) is the fourth leading cause of cancer mortality in the United States [1]. The 5-year survival rate has remained at 3% to 5% for the past three decades [1]. At the time of diagnosis, approximately 80% of patients present with locally advanced or metastatic disease that is resistant to therapy, and the median survival time after diagnosis is less than 6 months (2,3). Therefore, there is a need for a better understanding of the molecular mechanisms underlying the pathogenesis and progression of PDAC to develop new therapeutic strategies for increasing survival rates.

The most frequently detected mutations in PDAC suggest the genetic profile for this disease [2]. The mutational activation of K-ras is the earliest event identified in pancreatic carcinogenesis and is detected in nearly 100% of PDAC cases; loss of p16 has been identified in approximately 95% of PDAC cases and occurs through homozygous deletion (40%), intragenic mutation coupled with loss of the second allele (40%), or promoter hypermethylation (15%) [3]–[5]. To recapitulate the molecular pathogenesis of this disease, several experimental animal models have been established recently to determine the functions of mutated K-ras and inactivated p16 in pancreatic tumorigenesis [6], [7]. Mouse models showed that activation of *K-ras* induced pancreatic intraepithelial neoplasm (PanIN) lesions. Deletion of *p16INK4A/p14ARF* greatly accelerated the malignant progression of mutant K-ras-triggered PanIN lesions into highly invasive or metastatic PDAC [6]–[8]. These results suggest that activation of K-ras serves to initiate premalignant PanIN lesions and the p16/INK4A/p14ARF tumor suppressors normally function to inhibit the malignant transformation potential of mutant K-ras. However, human cancers are different in some aspects from murine cancer models as human cells are more resistant to both immortalization and malignant transformation than rodent cells [9], [10].

Only two immortalized and nontumorigenic pancreatic epithelial cell lines, human papilloma virus (HPV) E6E7-immortalized human pancreatic ductal epithelial (HPDE) and hTERT-immortalized human pancreatic epithelial nestin-expressing cell line (HPNE) cell lines were reported [11]–[13]. These two cells-based models were utilized for studying the mechanisms of human pancreatic cell tumorigenic transformation [14], [15]. Recently Leung et al. and our group reported that combination of the K-ras^G12D^ and inactivated Smad4 is sufficient to induce transformation of HPDE cells [16], [17]. Another recent study described a model of malignant transformation developed from HPNE cells through sequential introduction of HPV-16 E6E7, K-ras^G12D^, and the SV40 small t antigen. The transformed cell lines formed subcutaneous tumors in nude mice [18]. However, these models are more difficult to study mechanisms of molecular carcinogenesis in the human pancreas because the viral oncogenes used in this study are not associated with human PDAC development. Therefore, to recapitulates human pancreatic carcinogenesis and further explore mechanisms of tumorigenesis in pancreas without using unrelated viral oncogenes, several studies utilized HPNE cells to study the altered signaling pathways in PDCA development [19]–[21]. For example, Bera et al. showed that K-ras^G12D^ and loss of Smad4 cooperate to induce the expression of EGFR and to promote invasion, suggesting a potential mechanism of how a combination of oncogenic K-ras and loss of Smad4 leads to invasion [20].

Activated K-ras and inactivated p16 play an important role in human PDAC development. However, how these two genetic alterations act in concert to induce tumorigenic transformation in human pancreatic cells remains to be further explored. Here, we describe the establishment of a HPNE cell model expressing K-ras^G12D^ and Kras^G12D^/p16shRNA. We found that the expression of p16 was induced by K-ras^G12D^ in HPNE cells and that silencing the p16 expression induced by mutant K-ras in these cells resulted in tumorigenic transformation and development of PDAC in an orthotopic xenograft mouse model.

## Materials and Methods

### Cell lines and cell culture

The previously described [13] HPNE cell line was obtained from Dr. James W. Freeman at The University of Texas Health Science Center at San Antonio (Texas). HPNE cells were grown in Medium D with a mixture of M3 medium and DMEM containing one volume of M3 Base F culture medium (InCell Corp., San Antonio, USA), three volumes of glucose-free DMEM, 10% FBS, 5.5 mM glucose, 10 ng/ml EGF, and 50 µg/ml gentamycin. The 293T cell line was grown in complete DMEM. HPDE/K-ras cells were grown in keratinocyte serum-free medium.

### Antibodies and reagents

Antibodies to phospho-p38 MAPK, p38 MAPK kinase, Rb, pRb (S807/811), p14ARF and p15 were purchased from Cell Signaling Technology (Beverly, MA); antibodies to c-K-ras, Pan-ras, and p21 were purchased from Calbiochem (Gibbstown, NJ). Antibodies to p16, cytokeratin-19, Erk1/2, p-Erk, cyclin B1, cyclin E, c-Myc, matrix metalloproteinase 2 (MMP2), and urokinase plasminogen activator (uPA) were obtained from Santa Cruz Biotechnology (Santa Cruz, CA); Ras assay reagent and anti-human p27 were obtained from Upstate Biotechnology (Lake Placid, NY). Propidine iodide and gemcitabine were purchased from Sigma-Aldrich Chemical Co (St. Louis, MO); D-Luciferin was obtained from GoldBio Technology Inc (St. Louis, MO).

### Plasmid construction, transfection, retroviral and lentiviral production, and establishment of stable cell lines

Human mutant K-ras^G12V^ and p16shRNAs were cloned into retroviral vector PLHCX (Clontech Laboratories, Mountain View, CA) and lentiviral vector pLKO.1 (Addgene, Cambridge, MA), respectively. Retroviral K-ras^G12V^ vectors and the corresponding empty vector for generation of control cells, together with amphotropic packaging vector pCL-Ampho were transfected into 293T cells. The sequence of shRNA against human p16 and p14 is AACCATGCCCGCATAGATGCC. The shRNA oligos were cloned into lentiviral vector pLKO.1-TRC cloning vector (Addgene), and the puromycin cassette in p16p14shRNA/pLKO.1 was replaced with a zeocin resistance gene. Lentiviruses were generated by transfecting lentiviral vectors expressing p16p14shRNA/pLKO.1 together with packaging vector psPAX2 and envelope plasmid pMD2.G into 293T cells (gifts from Dr. Dihua Yu, MD Anderson Cancer Center). After 48-72 h transfection of retroviral or lentiviral vectors, viral culture supernatants from 293-T cells were used to infect the exponentially growing HPNE target cells at 50% confluence in the presence of 8 µg/mL polybrene (Sigma-Aldrich). After 48-72 h of infection, infected HPNE cells was selected with 300 µg/mL hygromycin B (Roche Diagnostics) and 400 µg/mL zeocin (Invitrogen) to purify polyclonal-infected populations of K-ras and p16shRNA expressing cells. The antibiotics-resistant clones of HPNE cells were pooled to establish HPNE/K-ras and HPNE/K-ras/p16shRNA cell lines and their respective control cell lines. The effects of knockdown were examined by Western blot analysis.

### Flow cytometric cell cycle analysis

HPNE cells were cultured in complete DMEM growth medium overnight, after starvation for 24 h in serum-free medium, then stimulated with complete growth medium for 12 h. Cells were harvested and cell cycle profiles of HPNE cells were analyzed by flow cytometry following FlowJo software analysis. Each experiment was performed independently three times with similar results each time. Results are expressed as the means ± s.d. of three independent experiments.

### Western blot analysis

HPNE cells were harvested from 10-cm dishes, cells were lysed in radioimmunoprecipitation assay protein lysis buffer, and 50 µg of each protein extract was subjected to analysis. The Western blot analysis was performed as previously described [22].

### Ras activity assay

The activity of the Ras protein was assayed by using the Ras assay reagent (Upstate Biotechnology) according to the manufacturer's instructions. HPNE cells were lysed with Mg-containing lysis buffer. Precipitation with Raf-1 RBD agarose and Western blot analysis were performed as described previously [14].

### Real-time PCR

Total RNA was extracted from the cells by applying TRIzol according to the manufacturer's protocol (Invitrogen). RNA quality and quantity were measured by an ND-2000 spectrophotometer (Nanodrop). cDNA was synthesized using the PrimeScript RT Master Mix (Bio-Rad). cDNA (20 ng) was subjected to real-time PCR performed with SYBR reagents using the IQ5 PCR system (Bio-Rad, Hercules, CA). β-Actin was used as the internal control gene. Specific primers were synthesized by Sigma-Aldrich and the sequences were described as follows: K-ras: forward primer: 5′-agagaggcctgctgaaaatg-3′, reverse primer: 5′-agtctgcatggagcaggaaa-3′; p16: forward primer: 5′-cccaacgcaccgaatagtta-3′, reverse primer: 5′-accagcgtgtccaggaag-3′; HBP1: forward primer: 5′-tcatcaccattggaaggagga-3′, reverse primer: 5′-ttgcaccatcccaaatcatca-3′;CHEK1: forward primer: 5′-atatgaagcgtgccgtagact-3′, reverse primer: 5′- tgcctatgtctggctctattctg-3′; CCNA1: forward primer: 5′-gaggtcccgatgcttgtcag-3′, reverse primer: 5′- gttagcagccctagcactgtc-3′; CDK2: forward primer: 5′- gtacctcccctggatgaagat-3′, reverse primer: 5′- cgaaatccgcttgttagggtc-3′; RFC1: forward primer: 5′- tggagaggcagttgcatgaag-3′, reverse primer: 5′- cctttcgagcctttttggtct-3′; MCM3: forward primer: 5′- tcagagagattacctggacttcc-3′, reverse primer: 5′- tcagccggtattggttgtcac-3′; β-Actin: forward primer: 5′-catgtacgttgctatccaggc-3′, reverse primer: 5′-ctccttaatgtcacgcacgat-3′.

### Senescence-associated β-galactosidase activity

To measure the Senescence-associated β-galactosidase (SA-β-gal) activity, HPNE cells were fixed with 2% formaldehyde/0.2% glutaraldehyde in PBS for 5 min, washed with PBS, stained with X-gal staining solution (1 mg/ml X-Gal, 5 mM potassium ferrocyanide, 5 mM potassium ferricyanide, 150 mM NaCl, and 2 mM MgCl_2_ at pH 6.0) at 37°C for 12–16 h. Cells were examined and a representative field of each experiment photographed at 15× magnification under the Olympus IMT-2 phase contrast microscope. Results are shown as percentage of SA-β-gal positive cells from three independent experiments.

### Cell proliferation assays

For the cell growth curve assay, HPNE cells were plated in 24-well plates (1×10^4^ cells/well) in triplicate in growth medium. Cells were counted at days 1, 3, 5, 7, and 9. Results were expressed as the means ± standard error of the mean from three independent experiments. For anchorage-independent growth assay, HPNE cells in 0.3% agarose with complete growth medium were seeded into 12-well plates (5×10^4^ per well) over a bottom layer of 0.6% low-melting-point agarose/growth medium and allowed to grow for 3 weeks. Colonies ≥0.2 mm in diameter were counted using the Olympus phase contrast microscope, and a representative field for each experiment was photographed at 15× magnification. At least three independent assays were performed in triplicate. Data were shown as the mean value of the number of colonies/per field ± the standard error of the mean from three independent experiments.

### siRNA transfection

HPNE/K-ras cells were seeded in 6-well plates (1×10^5^ cells/well) and transfected by using DharmaFECT transfection reagent (Dharmacon, Lafayette, CO) with 20 nM siRNA targeted against HBP1 or a scrambled RNA negative control according to the manufacturer's instructions. Cells were transfected for 48 h, and the expression of HBP1 and p16 was detected by real-time PCR.

### Orthotopic mice tumorigenicity assays

A total of 2×10^6^ viable HPNE cells in 50 µl of complete growth medium with 50% growth factor- reduced matrigel were injected into the pancreas of 4 to 6 week-old female non-obese diabetic/severe combined immunodeficient (NOD/SCID) mice; a total of five mice were used for each experimental condition. The condition of mice was monitored three times weekly over a period of 6 months. For in vivo imaging, an enhanced GFP/firefly luciferase double-expressing cassette FG12 was introduced into HPNE cells by lentiviral infection. The *in vivo* tumor growth was monitored in real time using the Xenogeny *In Vivo* Imaging System (Alameda, CA). As the mice tumor burden increased, mice were killed immediately upon becoming moribund, as required by our institutional animal care guidelines. After each mouse was killed, its tumor and some organs were removed and processed for routine histological analysis. Our animal protocol was approved by the MD Anderson Cancer Center Institutional Animal Care and Use Committee.

### Histological analysis of tumors

Mouse pancreas, liver, spleen, and tumors were fixed in 10% formalin and embedded in paraffin, and sections were stained with H&E according to standard procedures. The H&E slides from mouse xenografts were reviewed by a pathologist (Dr. Huamin Wang), who classified the tumor type and evaluated the tumor for necrosis and metastasis to other organs. A representative field for each histological type was photographed using an Olympus BX-51TF microscope.

### Statistical analysis

All data are presented as mean ± SD. For comparison of the differences among more than two groups, one-way ANOVA and Newman Keul's multiple comparison tests were used. All other comparisons were evaluated by using the Student' unpaired *t*-test (Prism GraphPad, San Diego, CA). A *P* value of <0.05 was considered statistically significant.

## Results

### Stable expression of mutant K-ras and p16shRNA in HPNE cells

**Figure 1:**
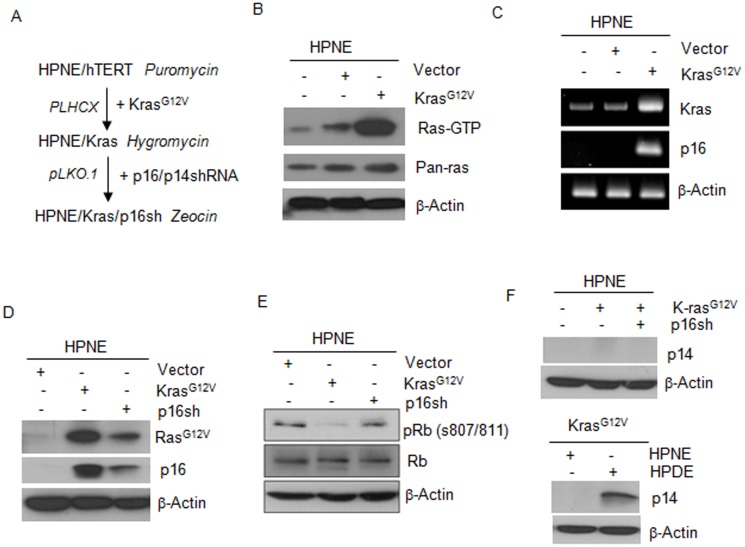
Mutant K-ras and p16shRNA were stably expressed in HPNE cells. (A) The strategy for introducing gene alterations into HPNE cell lines. (B) Stable expression of mutant K-Ras^G12V^ in the HPNE cell line, as identified by Ras activity assay and Western blot analysis of total Ras expression. (C) The expression levels of K-ras and p16 in HPNE/K-ras cells were identified by RT-PCR analysis. (D) Stable expression of mutant K-ras and p16shRNA in HPNE cell lines. The stable cell lines were identified by Western blot analysis of the expression of Ras^G12V^, total Ras and p16. (E) Western blot analysis of p-Rb and Rb expression in HPNE cell lines. (F) Western blot analysis of p14 expression in HPNE cell lines.

To study the mechanisms of tumorigenic transformation in human pancreatic epithelial cells, we sequentially introduced mutant K-ras, the earliest mutation identified in PDAC, and down-regulated p16 expression, another common alteration in PDAC, into an HPNE cells [13], [23]. The cells by retroviral and lentiviral vectors expressing mutated K-ras (K-ras^G12V^) and p16 shRNA respectively, to establish pooled antibiotic-resistant clones as cell lines (Fig.1A). Ras activity assay showed that expression of K-ras^G12V^ in HPNE cells induced higher levels of Ras activity than in vector-transfected control HPNE cells (Fig. 1B), a finding that was confirmed by RT-PCR analysis of K-ras mRNA and Western blot analyses of K-ras^G12V^ protein (Fig. 1C & 1D). We found that the expression level of p16 was not detectable using RT-PCR and Western blot assays (Fig.1C & 1D), indicating inactivation of p16 in HPNE (parental) cells. However, the p16 expression was markedly induced by K-ras in HPNE/K-ras cells and that the K-ras-upregulated p16 expression was greatly reduced by p16shRNA in HPNE/K-ras/p16shRNA cells (Fig.1C & 1D). To clearly clarify the functional status/activity of p16 in HPNE cells, the expression level of phosphorylated (p)-Rb (S807/811) was determined by Western blot. Immunoblotting analysis revealed that the expression level of p-Rb at sites S807/811 was markedly decreased in HPNE/K-ras cells as compared with parental HPNE cells, and substantially increased in HPNE/K-ras/p16sh cells (Fig. 1E), which was consistent with the expression level of p16 in those cells. Because the *p14ARF* gene is inactivated in 40% of PDAC, we designed a shRNA targeting the third exon of the *INK4A* locus common to both *p16* and *p14ARF t*o silence the expression of both p16 and p14. In cells expressing this shRNA, p14 protein expression was too low to be detectable in Western blot analysis using HPDE/K-ras as a positive control (Fig.1F). To rule out the possibility of cell cross-contamination, we performed DNA fingerprinting and found that the DNA fingerprint profile of the HPNE/K-ras/p16shRNA cell line did not match any known DNA fingerprints, but exactly matched that of the original source HPNE cell line (data not shown) [13], [23]. We thus successfully established HPNE cell lines with stable expression of the mutant K-ras and inactivated p16.

### Activation of K-ras and silencing of p16 in HPNE cells increased cell proliferation and growth

**Figure 2:**
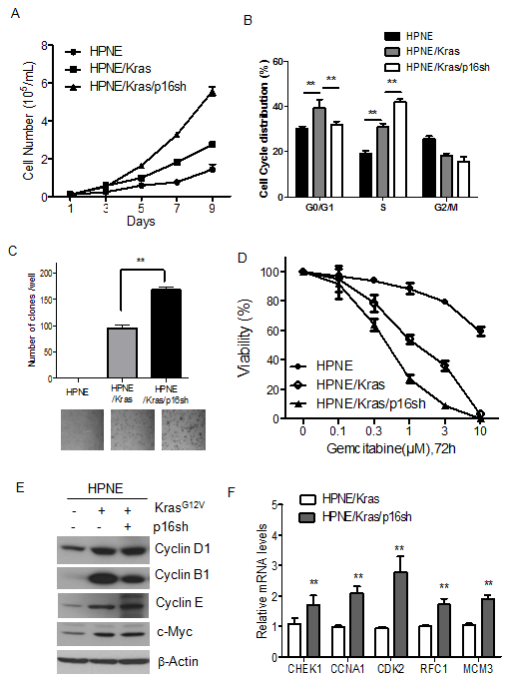
Activation of K-ras and silencing of p16 in HPNE cells increased cell proliferation and growth. (A) The cell growth curve of HPNE cell lines as detected by using a cell counter. (B) Cell cycle analysis of HPNE cell lines. The percentage of cells in each phase of the cell cycle is shown. (C) Anchorage-independent cell growth of HPNE cell lines in soft agar assay. A representative field of soft agar for each cell line is shown. (D) Cells were incubated with gemcitabine for 72 h, and cell viability was measured by MTT assay. (E) The expression of cell cycle proteins cyclins and c-Myc was increased by mutant K-ras in HPNE cell lines, as determined by Western blot analysis. β-Actin was used as loading control. (F) Relative mRNA levels of E2F target genes in HPNE/K-ras and HPNE/K-ras/p16sh cells. All data are presented as mean ± SD (n = 3 independent experiments). **P<0.01 versus the corresponding control groups.

Because uncontrolled cell growth is a hallmark of cancer, we first examined the growth features of HPNE/K-ras/p16sh cells. Neither HPNE/K-ras cells nor HPNE/K-ras/p16shRNA cells showed any significant morphological differences from control cells and original parental cells. Expression of mutant K-ras and knocking down of p16 in HPNE cells caused a marked increase in growth compared with control cell lines as determined by cell growth curve and cell cycle analysis (Fig. 2A & 2B). The percentages of cells in S phase for HPNE/K-ras and HPNE/K-ras/p16shRNA cell lines were 31.02±1.45% and 42.13±1.23%, respectively; both were higher than that of the control cells (19.1±1.27%; Fig. 2B).

To investigate whether HPNE cells had acquired the capacity for anchorage-independent growth, one of the hallmarks of *in vitro* cell transformation, we performed soft agar assays of HPNE/K-ras and HPNE/K-ras/p16shRNA cells. Unaltered HPNE cells did not form colonies, whereas mutant K-ras induced colony formation (Fig. 2C). Furthermore, the combination of mutant K-ras and p16 knockdown induced even more marked increases in the number and size of colonies (Fig. 2C), indicating that knocking down p16 increased the transformation potential of mutant K-ras. Additionally, MTT assays demonstrated that gemcitabine effectively inhibited cell growth in both HPNE/K-ras and HPNE/K-ras/p16sh cells in a dose-dependent manner, while HPNE cells were less sensitive to gemcitabine (Fig. 2D). These results suggest that the specific combination of expression of activated K-ras and knockdown of p16 is sufficient to transform HPNE cells *in vitro*.

As our results showed that mutant K-ras and p16 increased cell proliferation, we therefore examined the expression of cell proliferation related genes. We found that the expression of cyclin D1, cyclin E, cyclin B1, and c-Myc, which are involved in the regulation of cell cycle progression in the G1/S transition and the G_2_/M phases, as well as cell in proliferation and growth, was substantially induced by mutant K-ras in HPNE/K-ras and HPNE/K-ras/p16shRNA cell lines (Fig. 2E). Our previous data showed that loss of p16 increase the phosphorylation of retinoblastoma protein (Rb) (Fig.1E). Phosphorylation of Rb usually causes it to release the transcription factor E2F. We also measured mRNA levels of a representative number of E2F target genes known to function in cell cycle progression and DNA synthesis and replication. As shown in Figure 2F, nearly all the E2F targets investigated were expressed at significantly increased levels in HPNE/K-ras/p16sh cells. Taken together, these results suggest that both upregulation of cyclins and c-Myc by mutant K-ras, and activated E2F by p16shRNA may play important roles in increased cell proliferation and transformation.

### Activation of K-ras in HPNE cells increased cell invasion

**Figure 3:**
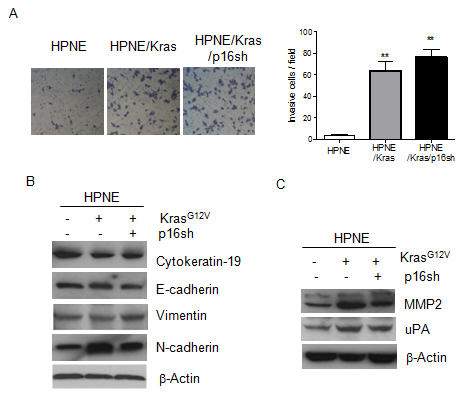
Activation of K-ras in HPNE cells increased cell invasion. (A) The quantification of stained HPNE cell lines per field in a transwell-matrigel penetration assay. Representative micrographs for each cell line are shown. Data are presented as mean ± SD (n = 3 independent experiments). **P<0.01 versus the HPNE control cells. (B) The expression of EMT markers cytokeratin-19, E-cadherin, vimentin, and N-cadherin was detected in HPNE cell lines by Western blot analysis. (C) The expression of invasion-related proteins MMP2 and uPA in HPNE cell lines was detected by Western blot analysis. β-Actin was used as loading control.

Since metastasis is also a hallmark of cancer, we next examined the invasive features of HPNE/K-ras/p16sh cells. Expression of mutant K-ras in HPNE cells significantly increased invasion of HPNE/K-ras and HPNE/K-ras/p16shRNA cell lines compared with control cell lines, as evidenced by an elevated capacity to invade through matrix gel in a transwell-matrigel assay (Fig. 3A). Because the transformation of HPNE cells increased their invasiveness, we then examined the expression of epithelial-mesenchymal transition markers and other invasion-related genes in HPNE cells. The expression levels of cytokeratin-19, E-cadherin, and vimentin were not substantially changed in the transformed cells (Fig. 3B). The expression levels of N-cadherin, MMP2 and uPA were induced by mutant K-ras and were markedly increased in HPNE/K-ras and HPNE/K-ras/p16shRNA cell lines (Fig. 3B & 3C). Our results suggest that upregulation of expression of N-cadherin, MMP2, and uPA contributes to tumorigenic transformation and metastasis of HPNE cells. Moreover, silencing of p16 seemed to have minimal effect on invasiveness on both cellular and molecular levels, suggesting that K-ras play a major role in the increased cell invasion in transformed HPNE cells.

### Inactivation of p16 enabled HPNE cells to overcome Ras-induced premature senescence

**Figure 4:**
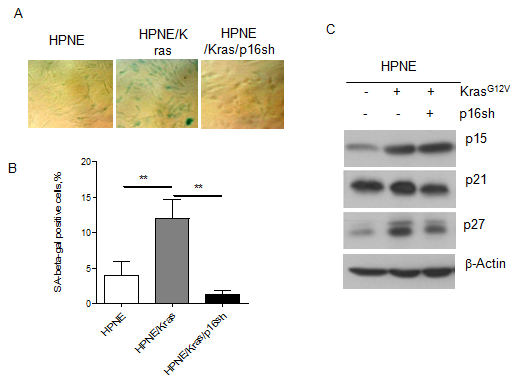
Knocking down of p16 enabled the HPNE/K-ras cells to overcome senescence. (A) Representative fields of senescence-associated β-galactosidase (SA-β-gal) activity in HPNE cell lines. (B) Quantification of SA-β-gal staining in HPNE cells, data are showed as percentage of positive cells **P<0.01 versus the corresponding control groups. (C) The expression of cyclin-dependent inhibitors – p15, p21 and p27 was analyzed by Western blot in HPNE cell lines. β-Actin was used as loading control.

Oncogene-mediated premature senescence has emerged as a potential tumor-suppressive mechanism in early cancer transitions. HPNE/K-ras cells with enhanced p16 expression displayed higher SA-β-gal activity than control cells; the percentage of HPNE/K-ras cells with SA-β-gal activity was higher than that of control HPNE cells. (Fig. 4A & 4B), suggesting that the activation of K-ras increased senescence in small fractions of HPNE/K-ras clones. K-ras–induced senescence was p16 dependent, as the silencing of K-ras–upregulated p16 expression abrogated senescence in HPNE/K-ras/p16sh cells (Fig. 4A & 4B). Hyperactivation of mitogenic stimulation may induce cyclin-dependent kinase inhibitors (CKI) such as p15, p21, p27. CKIs are strong inhibitors of cell proliferation and transformation. Also these CKIs play an important role in cell senescence and serve as significant markers associated with senescence. We also found that K-ras upregulated the expression of cyclin-dependent kinase inhibitors- p15, p21, and p27 in HPNE cell lines, but the expression of p21 and p27 in the transformed cell line HPNE/K-ras/p16sh was lower than that of the HPNE/K-ras cell line (Fig. 4C). These results indicate that expression of mutant K-ras and knockdown of p16 enabled the HPNE cell line to overcome senescence and acquire greater cell proliferation capacity.

### HPNE/K-ras/p16sh cells induced tumorigenesis and metastasis in NOD/SCID mice

**Figure 5:**
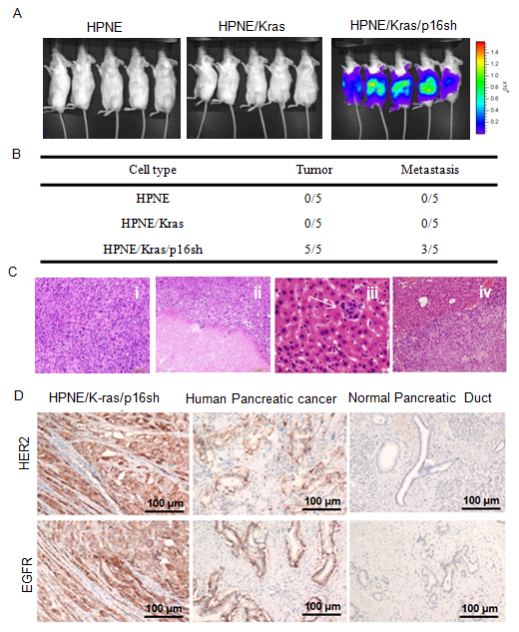
HPNE cells with activation of K-ras and inactivation of p16 induced tumorigenesis *in vivo*. (A) *In vivo* bioluminescence imaging of tumor growth by HPNE, HPNE/K-ras and HPNE/K-ras/p16shRNA cells at 8 weeks' after injection in NOD/SCID mice is shown. (B) The rates of tumor formation and metastasis in NOD/SCID mice. (C) Representative micrographs showing the histology of the orthotopic tumors formed by HPNE/K-ras/p16shRNA cells as revealed by H&E staining: (i) undifferentiated ductal carcinoma with sarcomatoid features, (ii) necrosis, (iii) liver metastasis, and (iv) spleen metastasis. (D) Immunohistochemical analysis of the expression of HER-2 and EGFR in tumors formed *in vivo* by the HPNE/K-ras/p16shRNA cells compared with those in human pancreatic cancer and human normal pancreatic duct. Scale bar: 100 µm.

We assessed the tumorigenic potential of HPNE, HPNE/K-ras, and HPNE/K-ras/p16shRNA cells *in vivo* using orthotopic tumorigenicity assays in NOD/SCID mice (Fig. 5). HPNE and HPNE/K-ras cell lines did not grow tumors in mice even after 6 months' observation (Fig. 5B), indicating that activation of K-ras alone in HPNE cells was not sufficient for tumorigenic transformation. However, tumor formation was observed in five/five mice injected with HPNE/K-ras/p16shRNA cells (Fig. 5A & 5B).

At eight weeks' after injection of HPNE/K-ras/p16shRNA cells, all mice had grown large tumors, become moribund, and been killed. Subsequent necropsy revealed their tumors to have irregular shapes and comprise multiple nodules. The tumors were highly aggressive, with metastases to the liver or spleen in three/five (60%) identified by either gross examination or H&E staining of sections of the liver and spleen (Fig. 5B & 5C). Histological analyses showed that the tumors were undifferentiated ductal carcinoma with sarcomatoid features (Fig. 5C). Tumors also displayed necrosis in many areas and many mitotic figures. The tumor cells had large pleomorphic nuclei and prominent nucleoli. HER-2 and EGFR are usually used as clinical markers for the diagnostic of pancreatic cancer. Using immunohistochemistry analysis, we also found that expression of HER-2 and EGFR was increased in the HPNE/K-ras/p16sh tumors compared with human normal pancreatic duct, similar to human pancreatic cancers (Fig.5D). Thus the combination of K-ras activation and loss of p16 in HPNE cells was sufficient to tumorigenically transform immortalized HPNE cells *in vivo* and induce tumorigenesis in NOD/SCID mice. These results suggest that activated K-ras and inactivated p16 play important roles in the development of human PDAC. Our results indicate that our model using human pancreatic epithelial cells closely mimics human pancreatic carcinogenesis.

### Molecular analysis of the mechanism of transformation

**Figure 6:**
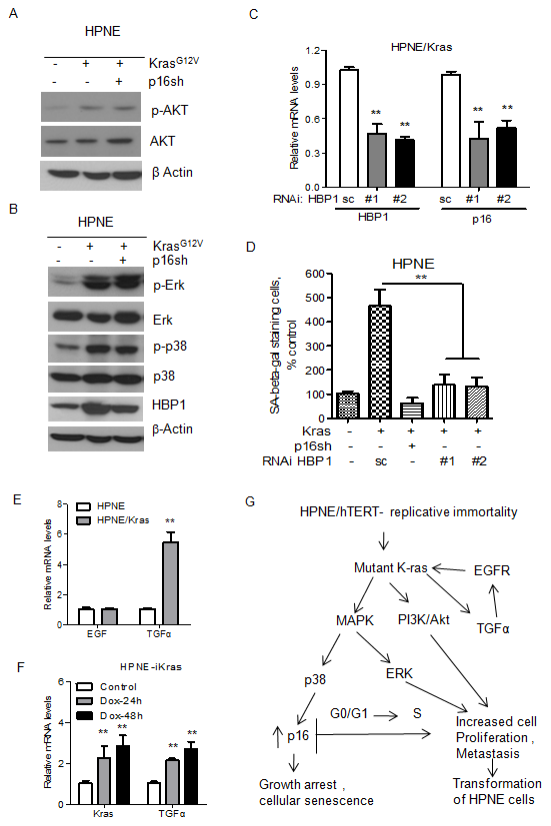
Molecular analysis of HPNE cell lines offers insight into malignant transformation in human pancreatic cancer. (A) Activation of the Akt signaling pathway by K-ras in HPNE cell lines as detected by Western blot analysis. (B) Activation of the MAPK signaling pathway by K-ras in HPNE cell lines. The expression of total and phosphorylated-Erk and p38 was detected by Western blot analysis. β-Actin was used as loading control. (C) Expression of p16 was decreased after siRNA depletion of HBP1, as detected by real-time PCR. (D) Relative quantification of SA-β-gal staining cells after siRNA depletion of HBP1 in HPNE/K-ras cells (E) Expression of EGF and TGFα was increased in HPNE/Kras cells as detected by real-time PCR. (F) Expression of TGFα was increased in HPNE-iKras cells as detected by real-time PCR. (G) The proposed model for transformation of HPNE cell line. hTERT enabled cell to acquire replicating immortality; oncogenic K-ras rendered HPNE cells able to sustain proliferative signaling, and thus increased cell proliferation and cell growth; and inactivation of p16 by shRNA silencing enabled HPNE cells to evade growth suppressors, disrupt the senescence checkpoint, and ultimately induce transformation of HPNE cells.

To dissect the molecular mechanisms of malignant transformation of HPNE/K-ras/p16shRNA cells, we first investigated the well-characterized downstream signaling pathways of K-ras, including the PI3K/Akt and MAPK pathways. Our results show that Akt was activated by activated K-ras in the HPNE/K-ras and HPNE/K-ras/p16shRNA cell lines compared with control cells (Fig. 6A). The MAPK pathway, Erk1/2 and p38 MAPK, was also activated by activated K-ras in the HPNE/K-ras and HPNE/K-ras/p16shRNA cell lines (Fig. 6B). Together, these results suggest that mutant K-ras activates its downstream Akt and MAPK pathways and that activation of these pathways may play critical roles in the transformation of HPNE cells.

Previous studies suggested that p38 MAPK activity leads to increased p16 expression regulated by transcriptional factor HMG box-containing protein 1 (HBP1) [24]–[27]. Consistent with these reports was our finding that the expression level of HBP1 protein was increased by activated K-ras in the HPNE/K-ras and HPNE/K-ras/p16shRNA cell lines compared with control cells (Fig. 6B). As expected, knockdown of HBP1 by siRNA decreased the p16 mRNA level and the percentage of Ras-induced premature senescence in HPNE/K-ras cells (Fig. 6C & 6D), indicating that HBP1 regulates p16 expression.

Two recent studies showed that development of K-Ras^G12D^-driven PDAC is totally dependent on EGFR signaling in genetic mouse models [28], [29]. To explore relationship among K-Ras, EGFR, and EGFR ligands in our HPNE model, we examined expression levels of EGFR ligands between HPNE and HPNE/K-Ras. The results showed that expression of TGFα was significantly increased, while EGF was not inducible in HPNE/K-ras cells (Fig. 6E). Consistently, the expression of TGFα was also increased in doxycycline-inducible HPNE-iKRas cells (Fig. 6F). Taken together, the reports for showing the requirement of EGFR for K-ras-induced PDAC [28], [29] and induction of TGFα by K-ras (Fig. 6E & 6F), there might be a feedforward signaling loop between TGFα and K-ras.

In summary, these results suggest that constitutively activated K-ras in HPNE cells activates the PI3K/Akt and ERK1/2 pathways to promote cell proliferation and growth; moreover, this K-ras activation can also trigger over expression of p16, leading to premature senescence through the p38 MAPK pathway, and silencing of p16 expression is required for tumorigenic transformation (Fig. 6G).

## Discussion

The present study demonstrates for the first time that the expression of mutant K-ras and the silencing of K-ras–upregulated p16 expression in HPNE cells together induce tumorigenic transformation. The transformed cells exhibited increased cellular proliferation and invasion *in vitro*, and upregulation of EMT markers and invasion-related proteins was involved in the transformation. The transformed cells also exhibited increased tumor growth in an orthotopic mouse model *in vivo*. Histological analysis of the tumors formed by the HPNE/K-ras/p16shRNA cell line revealed undifferentiated ductal carcinoma with sarcomatoid features with metastasis to liver and spleen. The histological features of these tumors are consistent with the results found in a previous mouse model of mutant K-ras and deletion of *p16INK4A/p14ARF* [6], [7], with a higher propensity for poorly differentiated PDAC histology with undifferentiated sarcomatoid features. Molecular analysis revealed activation of K-ras downstream Akt and MAPK signaling pathway. These results suggest that expression of mutant K-ras and knocking down of p16 were sufficient for tumorigenic transformation of immortalized human pancreatic cells into pancreatic cancer cells and that mutation of K-ras and loss of p16 play important roles in the tumorigenic transformation. This novel transformation model induces malignant transformation of human pancreatic epithelial cells with the most frequently identified genetic alterations in PDAC.

Recent progress in human cell-culture transformation models has shown that a variety of different normal human cells can be induced to malignant transformation through introduction of various combinations of genetic alterations [18], [30]–[35]. However, one striking difference between our previous and current studies is that the previous studies depended on the expression of DNA tumor viral oncogenes, such as the SV40 early region encoding both the large T and small t antigens and HPV E6E7 genes, to induce malignant transformation [18], [30]–[32]. These viral genes are not associated with most human cancers. Moreover, the cellular target and function of these viral genes in malignant transformation remain poorly defined, which makes the study of the mechanisms of transformation more complicated. Our model uses only the three most common gene alterations seen in PDAC: hTERT, mutant K-ras, and silencing of p16 to acquire immortality, sustained proliferative signaling, evasion of growth suppressors, disruption of the senescence checkpoint and ultimately, tumorigenesis, thus providing insights into the mechanisms of human pancreatic carcinogenesis.

Requirements for Ras expression level in transformation vary [31], [36]. Elenbaas showed that the tumorigenicity of human mammary epithelial cells by SV40 large T antigen, hTERT, and oncogenic H-rasV12 was dependent on the levels of Ras oncogene expression; high level of oncogenic Ras expression was required for transformation [31]. Another recent study demonstrated that increased Ras activity plays a crucial role in pancreatic tumorigenesis, expression of a high level of mutant K-ras in acinar cells using a transgenic method led to elevated Ras activity similar to that found in PDAC cells and caused mice to develop PDAC [36]. In contrast, other studies demonstrated that overexpression of mutant K-ras protein was not required for transformation. High levels of Ras expression may result in senescence [37]. There may exist a selection against cells that overexpress K-ras, leaving a population with moderate expression of activated K-ras to undergo transformation. In fact, lower levels of oncogenic Ras more closely approximate those observed in human tumors. In the HPNE cell transformation model, the Ras expression in transformed cells with mutant K-ras and knockdown p16 was lower than that of the K-ras expressing cell line, indicating that a high level of Ras expression is not required for transformation of HPNE cells.

p16 is a potent inducer of senescence in many cell types [38], [39]. The marked increase in expression of p16 induced by mutant K-ras in HPNE cells is consistent with an earlier report that, p16 expression in primary human fibroblasts was stimulated by Ras^V12^ [37], [40]. The upregulation of p16 expression in cells with mutant Ras is regarded as an important barrier to transformation. In fact, p16 plays a critical role in blocking the progression of K-ras–initiated pancreatic neoplasms into invasive or metastatic PDAC in mouse models [6], [7]. In our model, mutant K-ras moderately promoted cell proliferation and loss of p16 further increased growth in K-ras–expressing cells. Moreover, we found that silencing K-ras–upregulated p16 expression abrogated oncogenic K-ras–induced senescence in HPNE cells, suggesting that inactivation of p16 is a key mechanism required to disrupt the senescence checkpoint and trigger cell transformation in mutated K-ras cells. Previous report also showed that the mutated K-ras induces p16 hypermethylation, resulting in transcriptional silencing of INK4-ARF in human embryonic stem cells [41]. Epigenetic silencing through aberrant methylation of the p16 promoter overcomes the senescence barrier and is a decisive step to malignant transformation. However, we can't find any strong evidence to explain this contradictory effect of mutated K-ras on p16 expression. It may depend on different cell types or cell lines and worth studying in our future research.

The Ras effector PI3K/Akt and MAPK pathway play a pivotal role in Ras-triggered transformation and tumorigenesis. Our results show that Akt is highly activated in the transformed HPNE/K-ras/p16shRNA cell line, indicating that PI3K/Akt may play a critical role in Ras-driven tumorigenic transformation of HPNE cells. The MAPK pathway regulates diverse cellular programs by relaying extracellular signals to intracellular responses, mainly including the ERK1/2, JNK1-3 and p38 [24]. The ERK1/2 pathway has been associated with numerous processes including differentiation or proliferation. The p38 MAPK pathway has been extensively associated with apoptosis, but its role in cell cycle regulation, senescence, and tumor suppression has recently come under intense study [24]–[27]. Our results show that both the ERK1/2 and p38 MAPK pathways were activated by activated K-ras in the HPNE/K-ras and HPNE/K-ras/p16shRNA cell lines compared with control cells. Moreover, K-ras and p38 MAPK signaling engaged HBP1 and p16 to trigger premature senescence. Our present study data link HBP1 with p16, showing HBP1 increases p16 expression to regulate senescence in HPNE/K-ras cells. p16 acts as a functionally relevant player in a Ras/p38 MAPK/HBP1 senescence pathway to induce premature senescence in HPNE/K-ras cells. Whether HBP1 knockdown facilitates Ras-induced transformation and tumorigenesis is worthy studying in our future research. Additionally, the potential of a feedforward signaling loop between TGFα and K-ras^G12D^ is an interesting one and may provide insight into the requirement of EGFR for K-ras^G12D^-induced PDAC.

In conclusion, through the stepwise introduction of mutant K-ras and silencing of p16 genes into HPNE cells, we demonstrated that the expression of mutant K-ras and the silencing of K-ras–upregulated p16 expression in HPNE cells induced tumorigenic transformation, and thus developed a novel experimental cell culture model to recapitulate human pancreatic carcinogenesis. These findings enhance our understanding of the signaling pathways important for malignant transformation in PDAC. This is one of the experimental cell culture models for studying the mechanism of tumorigenic transformation of human pancreatic epithelial cells by utilizing the signature mutations found in PDAC for better revealing the mutation-altered signaling pathways in human pancreatic carcinogenesis.
