# Expression of Concern: Cooperativity of Oncogenic K-Ras and Downregulated p16/INK4A in Human Pancreatic Tumorigenesis

**DOI:** 10.1371/journal.pone.0225279

**Published:** 2019-11-08

**Authors:** The PLOS ONE Editors

After publication of this article [1], concerns were raised about similarities between the β-actin panels shown in Figures 1E and 4C. The authors disagree with this concern but the original blot images supporting these figure panels are no longer available. Similarities were also noted between the β-actin blots in Figures 2E and 3B, when one of these is rotated 180°.

In addition, in the “Stable expression of mutant K-ras and p16shRNA in HPNE cells” subsection of the Results, the authors referenced data not shown in discussing the DNA fingerprint profile of HPNE/K-ras/p16shRNA cell line as compared to the DNA fingerprints of parental HPNE and other cell lines: “To rule out the possibility of cell cross-contamination, we performed DNA fingerprinting and found that the DNA fingerprint profile of the HPNE/K-ras/p16shRNA cell line did not match any known DNA fingerprints, but exactly matched that of the original source HPNE cell line (data not shown).” The authors provided DNA fingerprinting data for HPNE cells as Supporting Information (S1 File); other data needed to support the statement are no longer available.

The primary data underlying results in this article were not included with the published article although the Data Availability Statement for this article stated, “The authors confirm that all data underlying the findings are fully available without restriction. All relevant data are within the paper.” With this notice, the authors provide the original raw data supporting parts of Figures 1–6 as Supporting Information (S1 File). The other data supporting the reported results are no longer available, including:

- blot/gel data for several Figure 1 panels, and for Figures 2E (β-actin), 3B (E-Cadherin, β-actin), 3C, 4C (p21, β-actin), 6A, 6B (Erk, HBP1, β-actin);
- original images supporting Figures 2C, 3A, 4A, 5Ciii, iv;
- individual-level data supporting graphs in Figures 3A, parts of Figure 2, and Figure 6.

The *PLOS ONE* Editors issue this Expression of Concern to notify readers of the data unavailability and the unresolved concerns about the β-actin panels discussed above.

## Supporting information

S1 File Underlying data provided for HPNE DNA fingerprinting and for the following figures: 1D (RasG12V, P16), 1E (pRb, Rb), 1F (P14), 2C (*error bars appear different in figure and PDF), 2E (c-myc, Cyclin E, CyclinB1, CyclinD1), 3B (Vimentin, Cytokeratin-19, N-Cadherin), 3C (uPA), 4B (*results in Figure appear different than those in the PDF), 4C (p15, p27, p21), 5A, 5Ci, ii, 5D, 6B (p-P38, p38, p-Erk).
(PDF)
