# Supplementary material for: Expression of Concern: Cooperativity of Oncogenic K-Ras and Downregulated p16/INK4A in Human Pancreatic Tumorigenesis
Source: PLoS One. 2019 Nov 8;14(11):e0225279. doi: 10.1371/journal.pone.0225279 (PMC6839839; doi:10.1371/journal.pone.0225279)
Supplement: S1 File — Underlying data provided for HPNE DNA fingerprinting and for the following figures: 1D (RasG12V, P16), 1E (pRb, Rb), 1F (P14), 2C (*error bars appear different in figure and PDF), 2E (c-myc, Cyclin E, CyclinB1, CyclinD1), 3B (Vimentin, Cytokeratin-19, N-Cadherin), 3C (uPA), 4B (*results in Figure appear different than those in the PDF), 4C (p15, p27, p21), 5A, 5Ci, ii, 5D, 6B (p-P38, p38, p-Erk). (PDF) [file pone.0225279.s001.pdf]

Attachment 2.

THE UNIVERSITY OF TEXAS
MD Anderson
Cancer Center
Making Cancer History®

Cell Line Cha

CCSG-Characterized Cell Line Core

CCLC database is a comprehensive databases of short tandem repeat (STR) profiles, The database includes profiles from 20 public database/ public
The database contains over 4000 unique human cancer cell line STR profiles, one of the largest cancer cell line STR online search database in the v

For general questions please contact Kathryn Aziz (Coordinator) (713)-792-5743 , KEHodges@mdanderson.org
For technical questions please contact Xuesong Li (Core research Investigator) (713)-792-6833 , Xsli@mdanderson.org
Core Director: Dr. Karina Eterovic (713)-792-5754, aketerovic@mdanderson.org

Core Grant Citation

This facility is funded by NCI # CA16672. Publications should cite the Core grant in the acknowledgment section, if publication
Please note that if you are to every publish these cell lines ***do not use all 14 loci/alleles on the paper, ONLY 8 loci/alleles can be u**

SET 423	2/26/18

Source	Sample_Name	AMEL	CSF1PO	D13S317	D16S539

Jianhua Ling	BxPC-3	X	13	11	9,11
DSMZ, ATCC ,JCRB, Riken online search database	BXPC3	X	13	11	9,11

Source	Sample_Name	AMEL	CSF1PO	D13S317	D16S539
Jianhua Ling	MIA-PaCa-2A	X	10	12,13	10,13
Public database_Sanger	MIA-PACA-2	X	10	12,13	10,13

Source	Sample_Name	AMEL	CSF1PO	D13S317	D16S539
Jianhua Ling	hTERT-HPNE	X,Y	12	12,13	12,13
Public database_DSMZ	HTERT-HPNE	X,Y	12	12,13	12,13

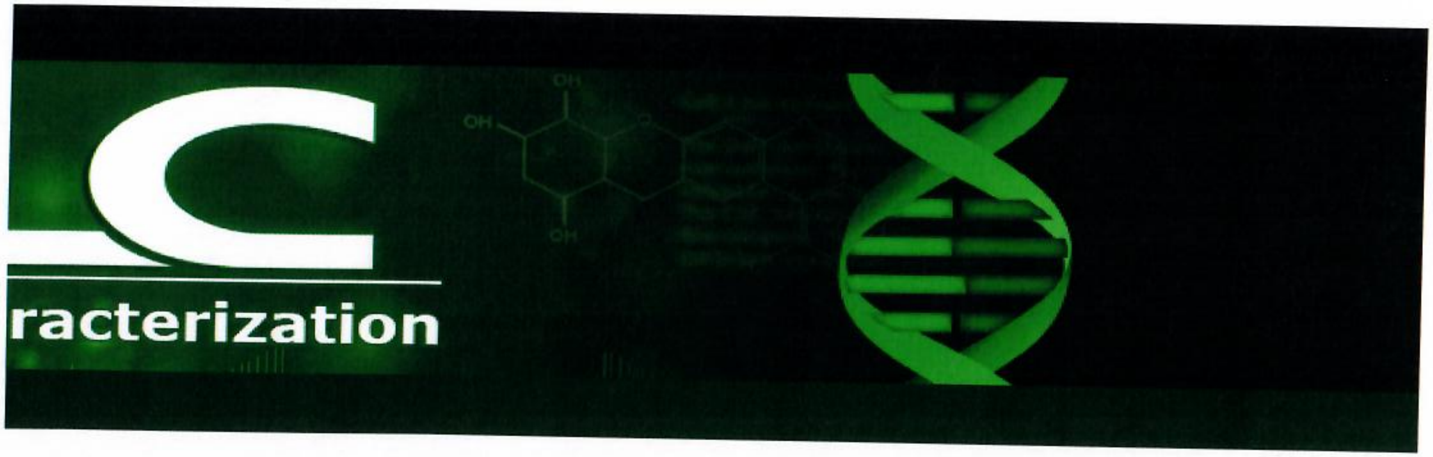

ations and the cell lines developed by MD Anderson research labs.
vorld

s use data generated by the Core facility. Two copies of the publication acknowledging the Core gra
ised...HIPPA & M. D. Anderson's policy*

D18S51	D21S11	D3S1358	D5S818	D7S820	D8S1179	FGA	TH01	TPOX	vWA

12	29	14,16	11	10,13	13	20,21	9	8	14,18
12	29	14,16	11	10,13	13	20,21	9	8	14,18

D18S51	D21S11	D3S1358	D5S818	D7S820	D8S1179	FGA	TH01	TPOX	vWA
12	29,31.2	16	12,13	12,13	16	22	9,10	9	15
12	29,31.2	16	12,13	12,13	16	22	9,10	9	15

D18S51	D21S11	D3S1358	D5S818	D7S820	D8S1179	FGA	TH01	TPOX	vWA
13,16	30	15	11	9,10	10,13	23,24	8,9	8,11	17
13,16	30	15	11	9,10	10,13	23,24	8,9	8,11	17

[redacted]

THE UNIVERSITY OF TEXAS
MD Anderson
~~Cancer~~ Center
Making Cancer History®

nt should also be submitted to the facility at Unit 950.

Comments

attachment3 for Fig2C

HPNE	HPNE/K-ra	HPNE/K-ra	HPNE/K-ra	HPNE/K-ra	HPNE/K-ra	HPNE/K-ras/p53mut
0	97.66667	154	134.6667	53.66667	69.33333	43
0	1.527525	7.211103	8.082904	4.725816	7.023769	6.082763

smad4sh	p53sh	HPNE/Kras/p53mut
50	70	50
59	62	40
52	76	39
53.66667	69.33333	43
4.725816	7.023769	6.082763

2C

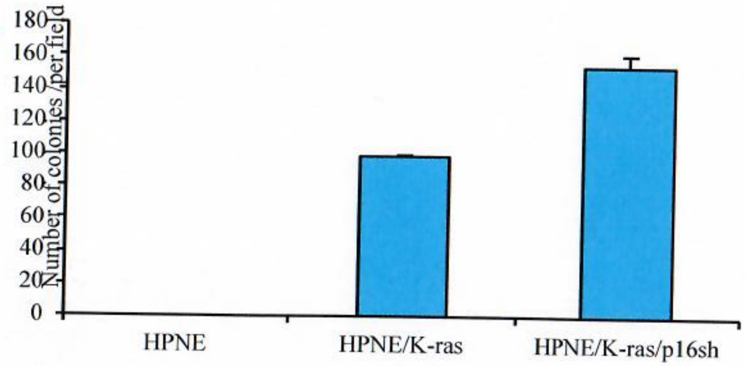

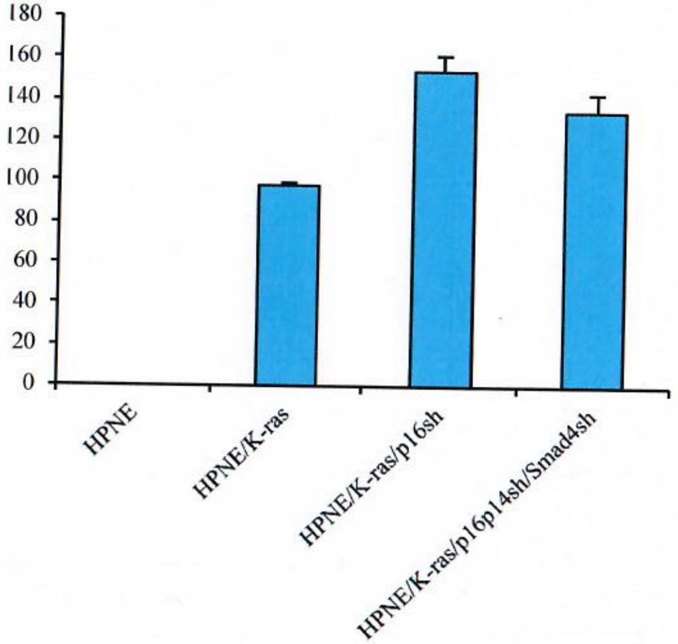

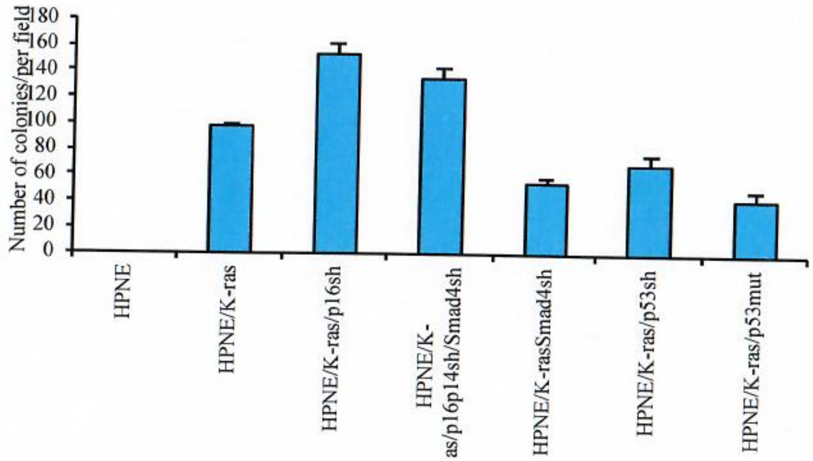

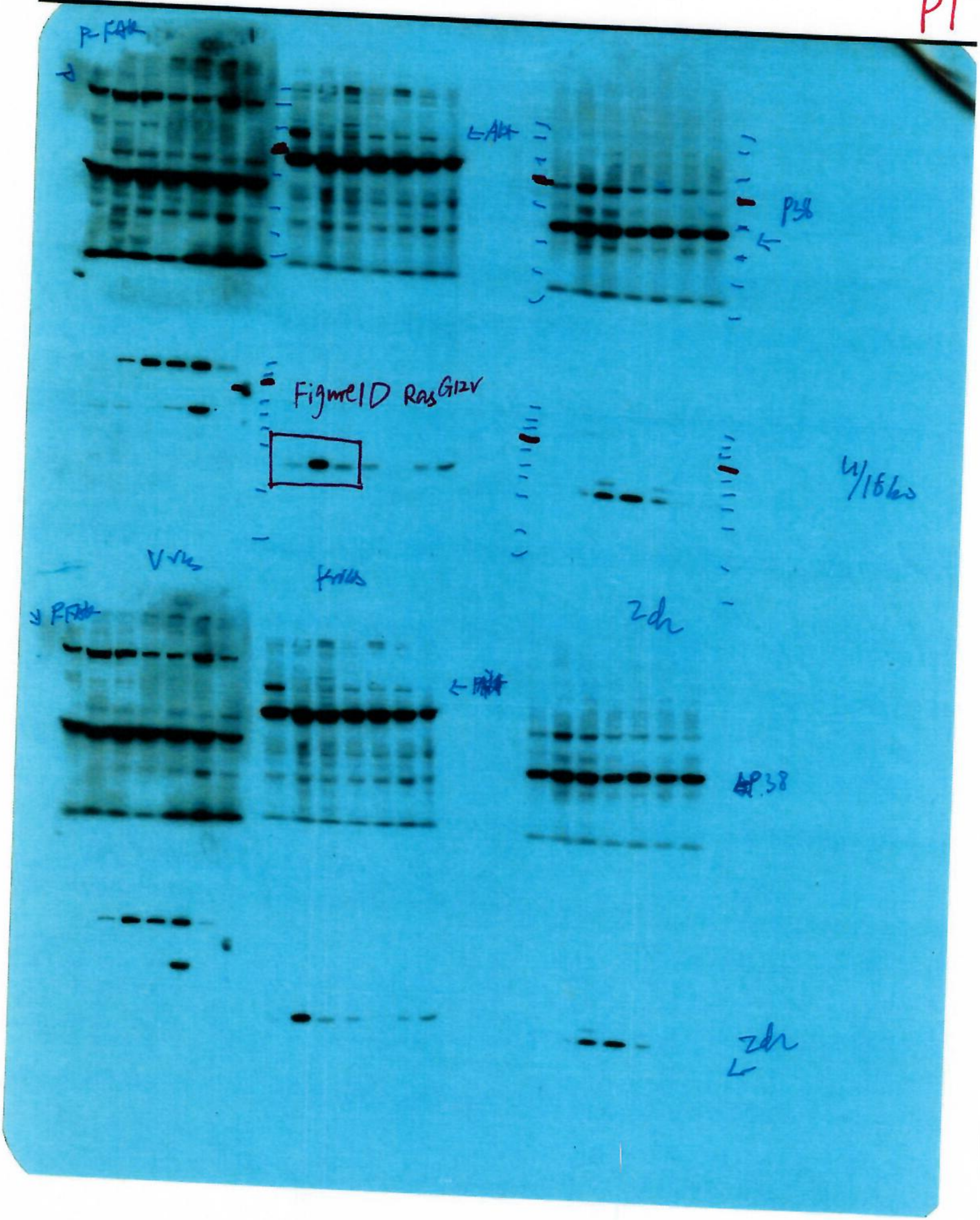
Figure1D RasG12V
P38

P2

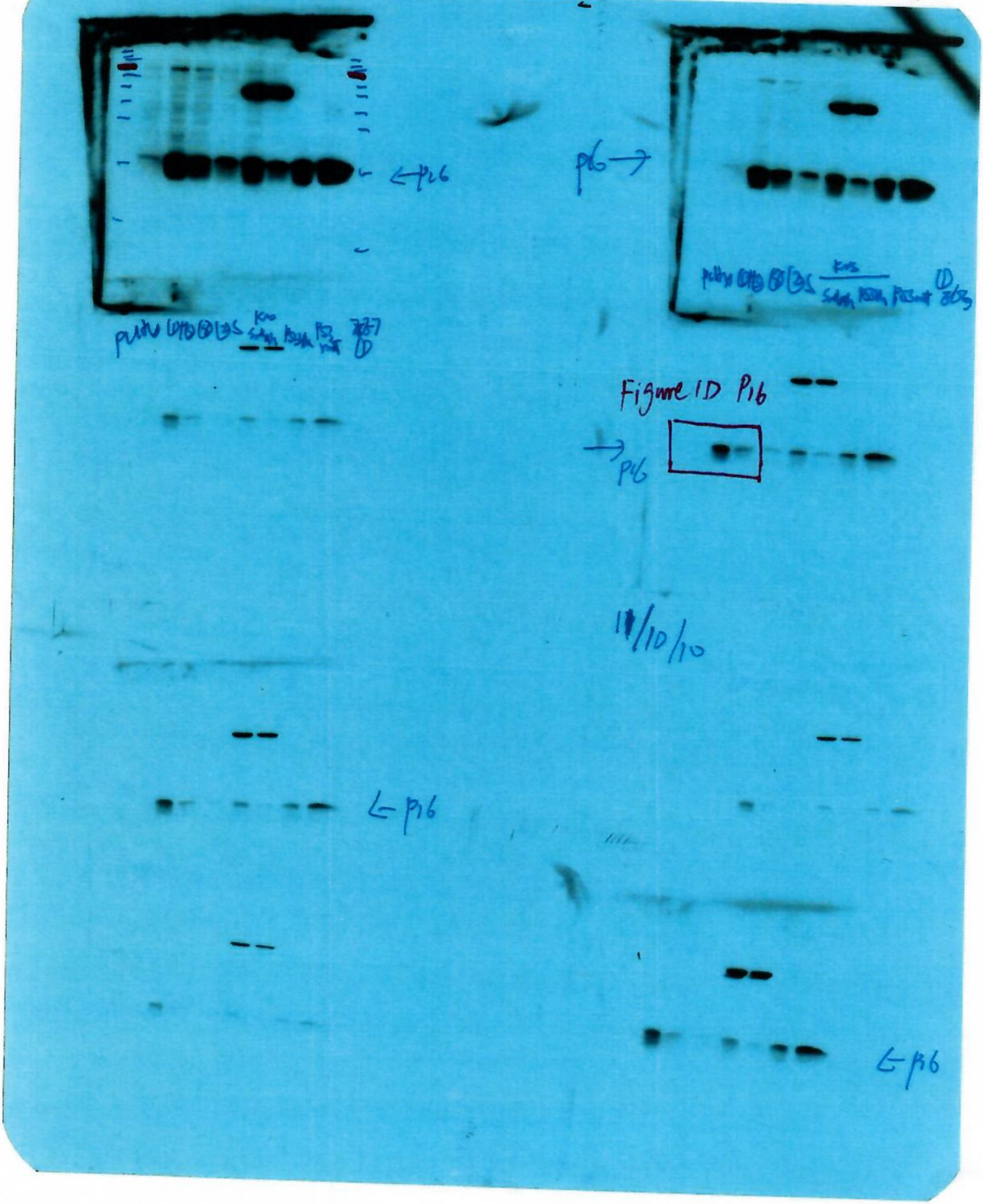

Figure 1E

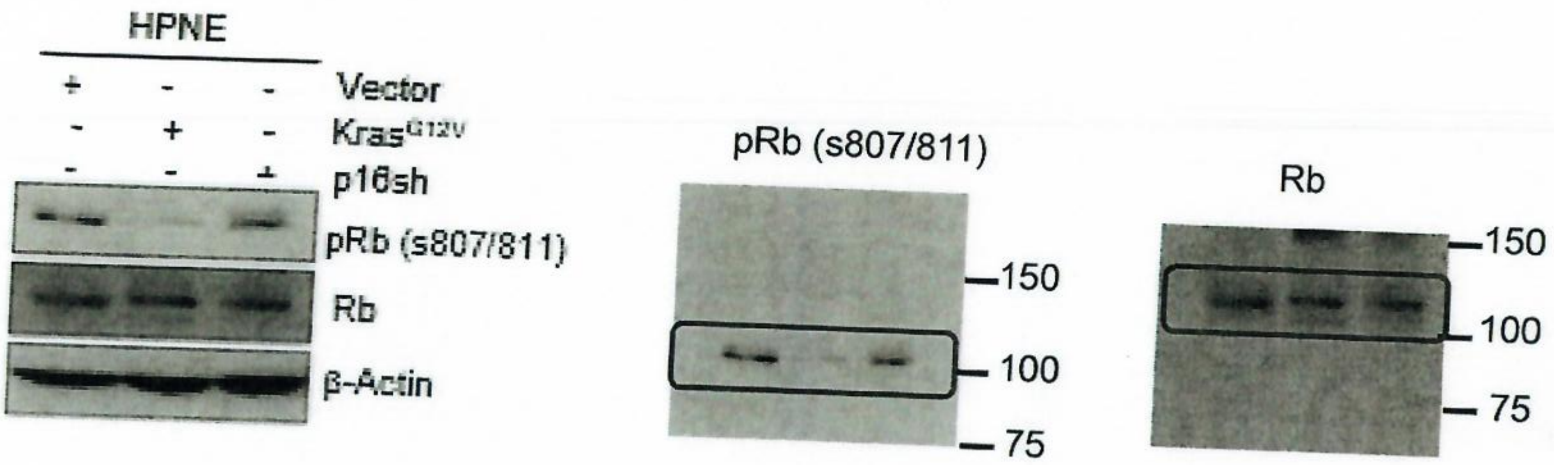

P2-2

P3

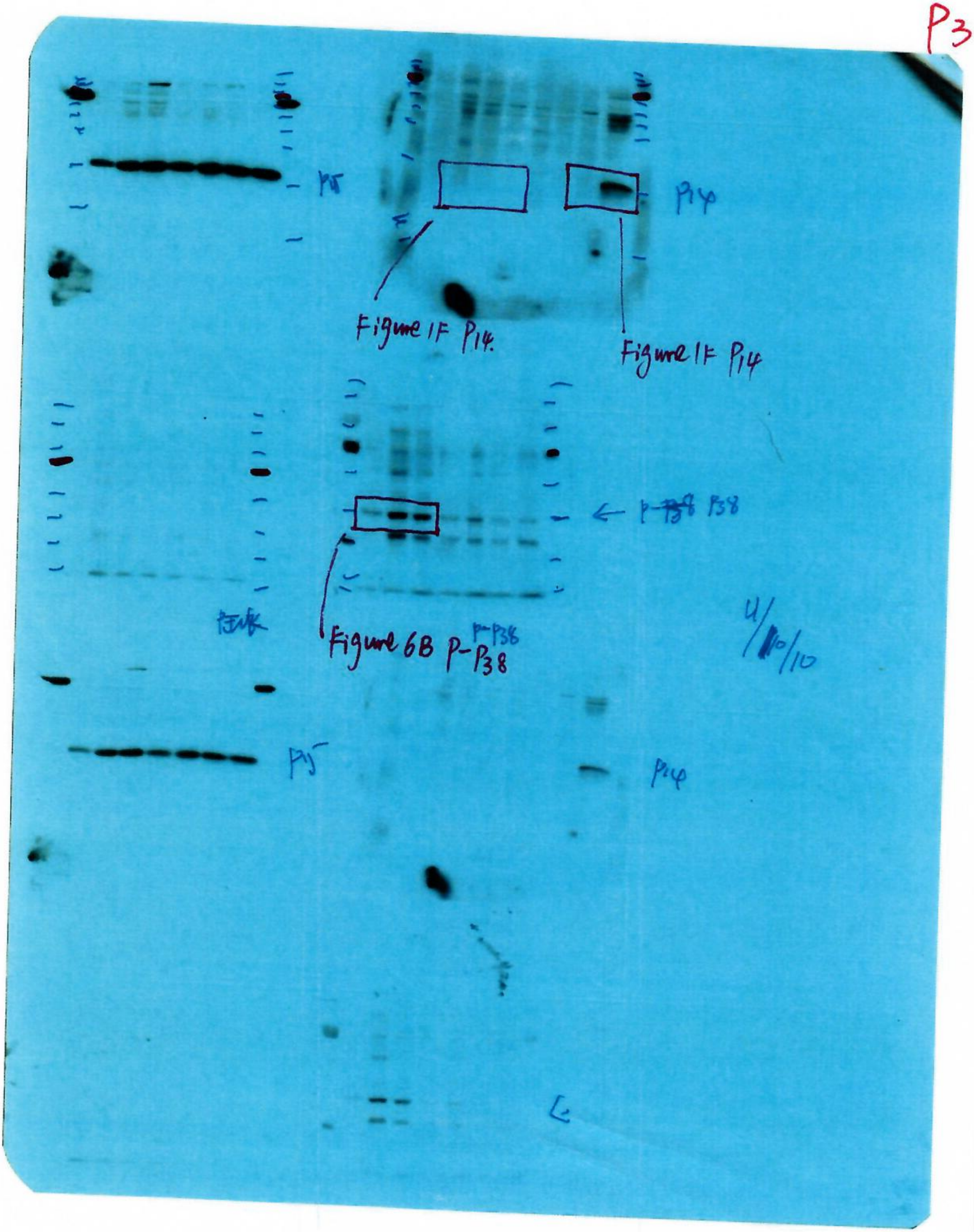
P15
P14
Figure 1F P14.
Figure 1F P14
P-P38
Figure 6B P-P38
11/10/10
P15
P14

P4

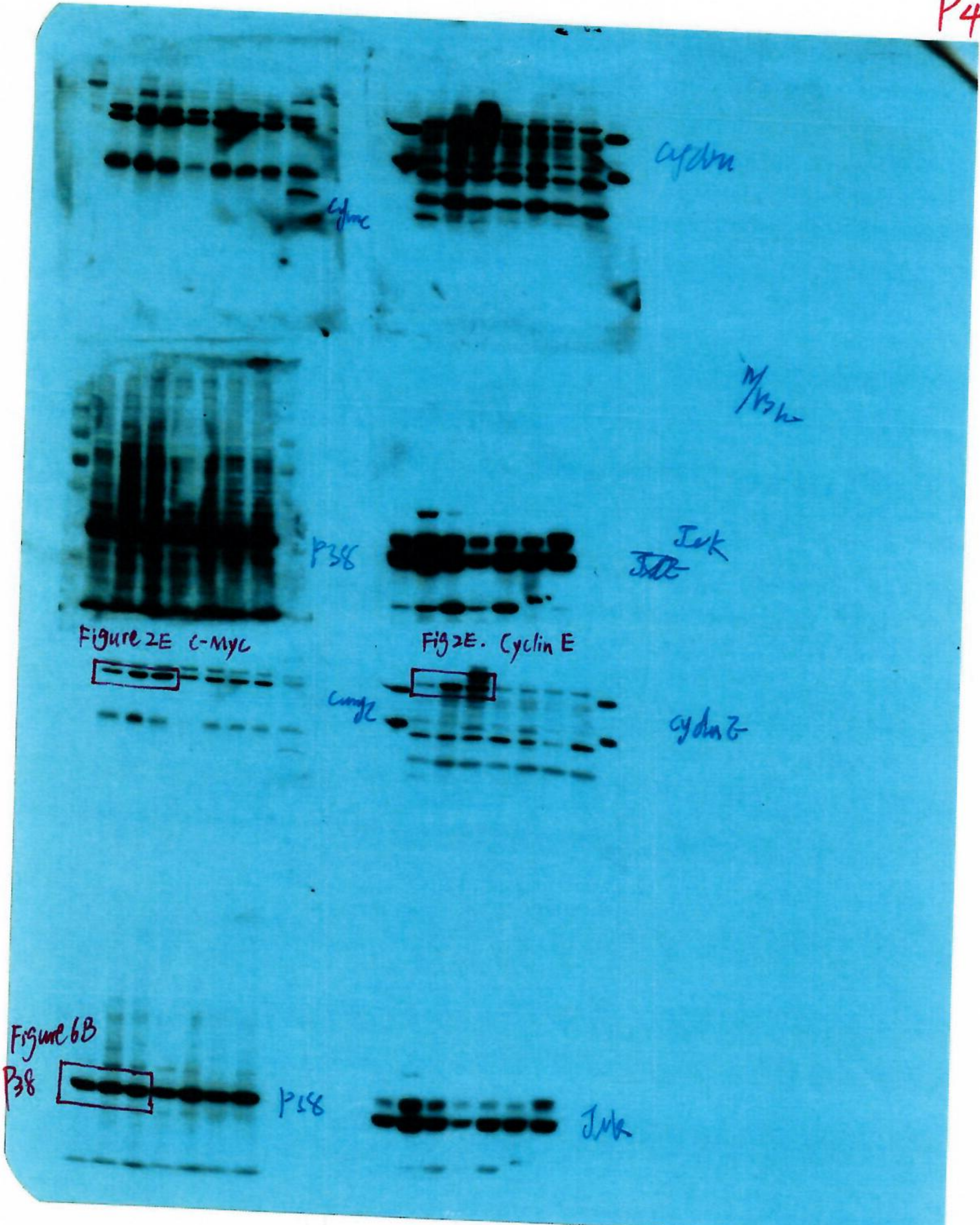

P5

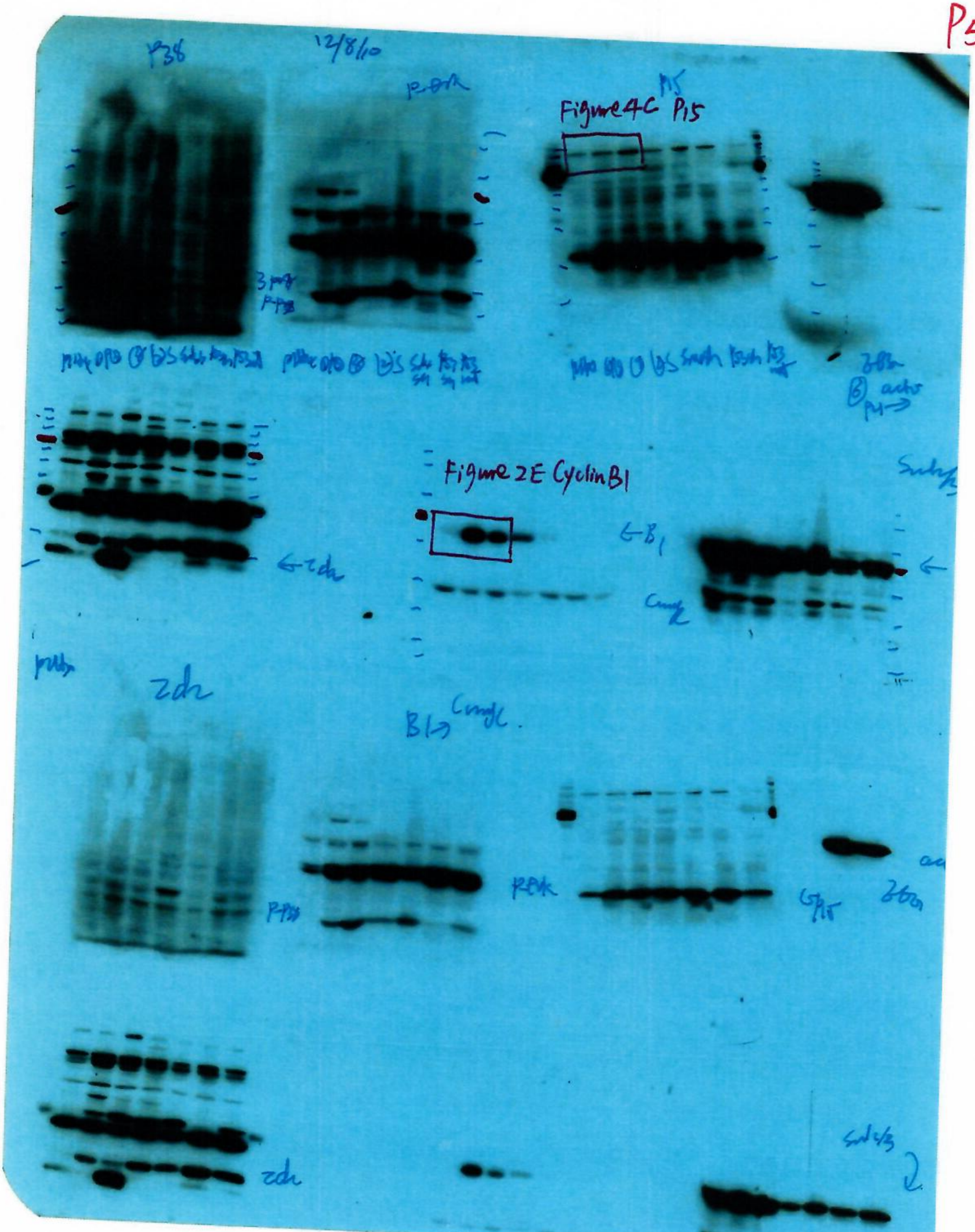

P6

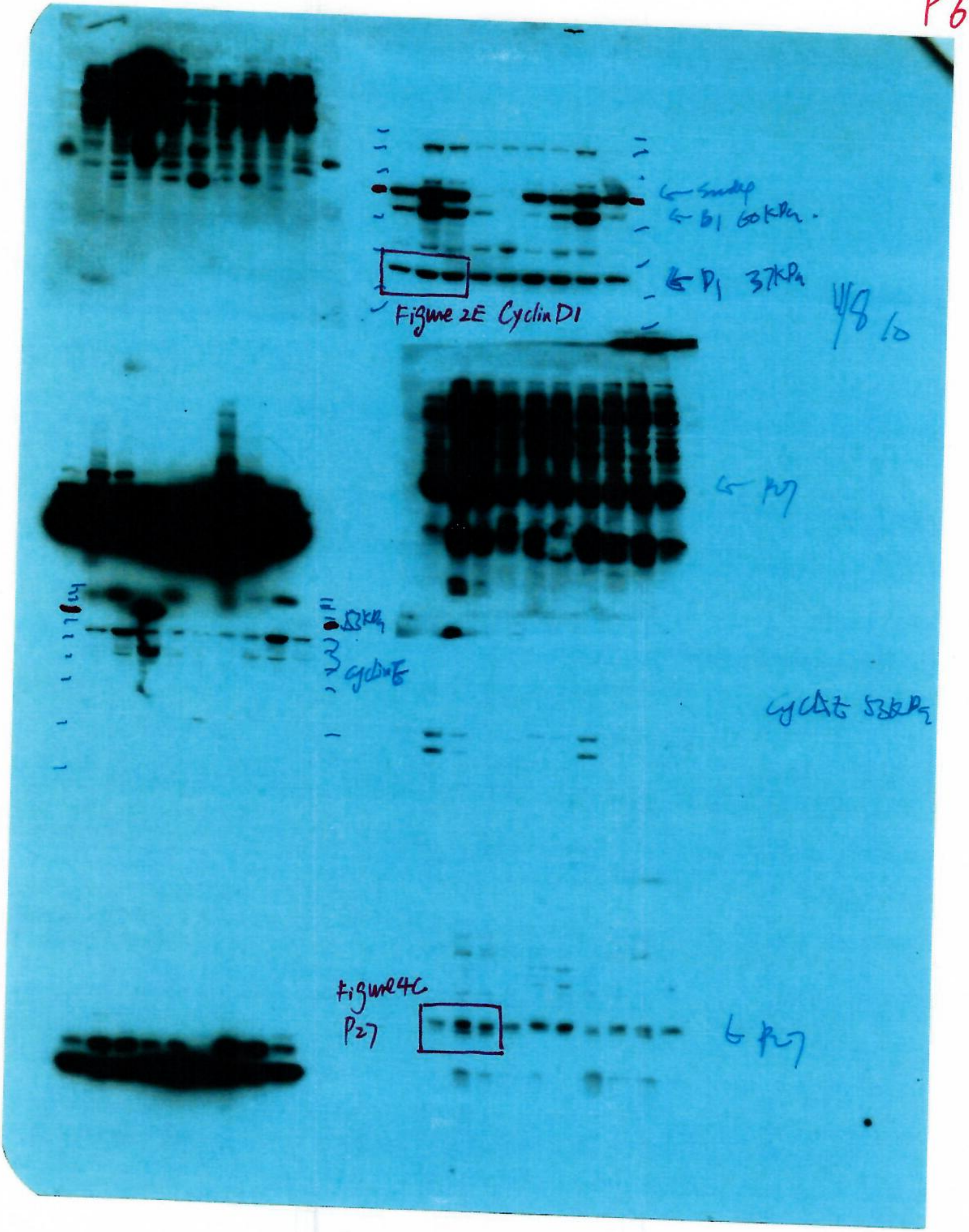
D1 60kDa
D1 37kDa
Figure 2E CyclinD1
4/8/10
p27
53kDa
cyclinE
Figure4C
P27
p27

P6-6

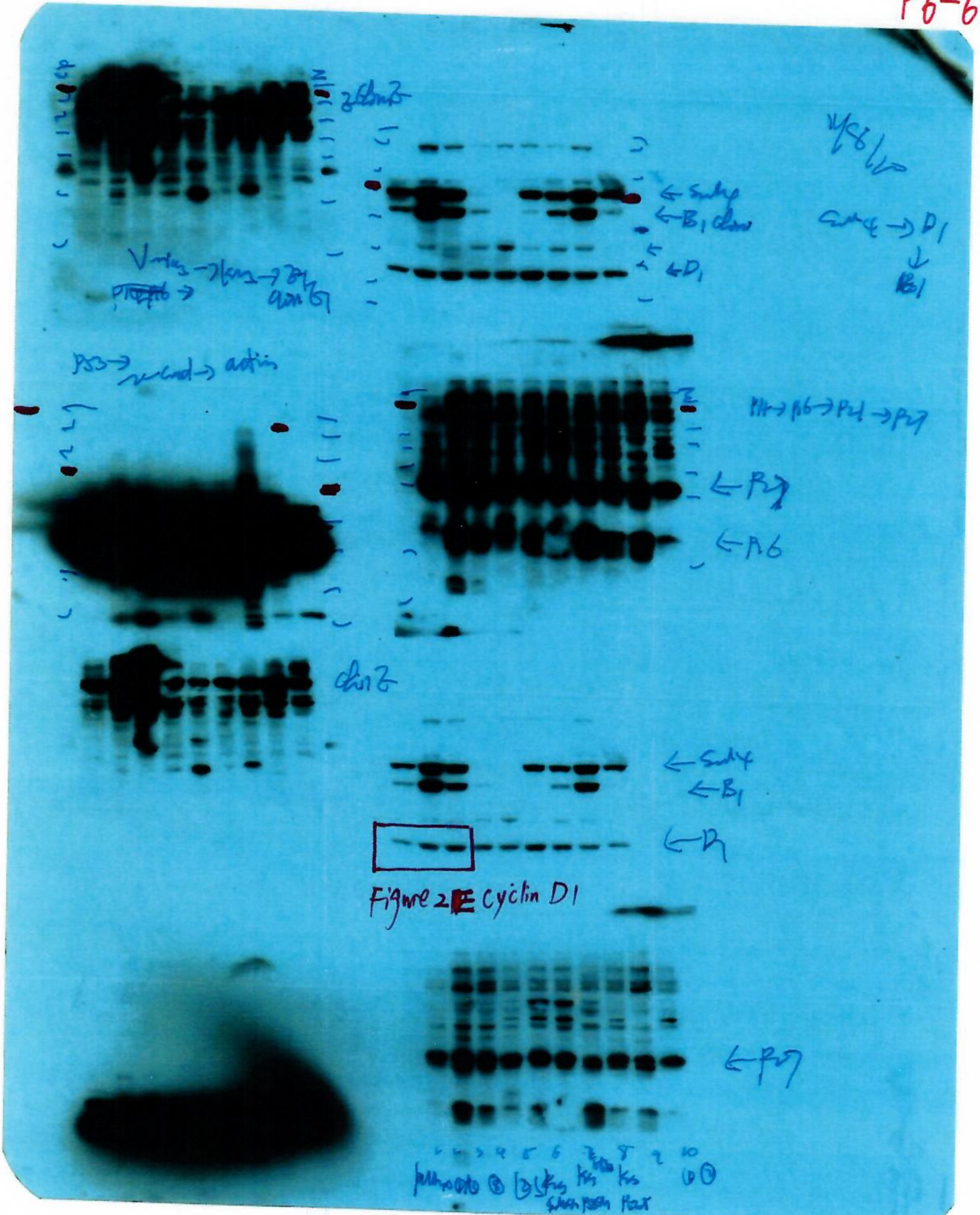

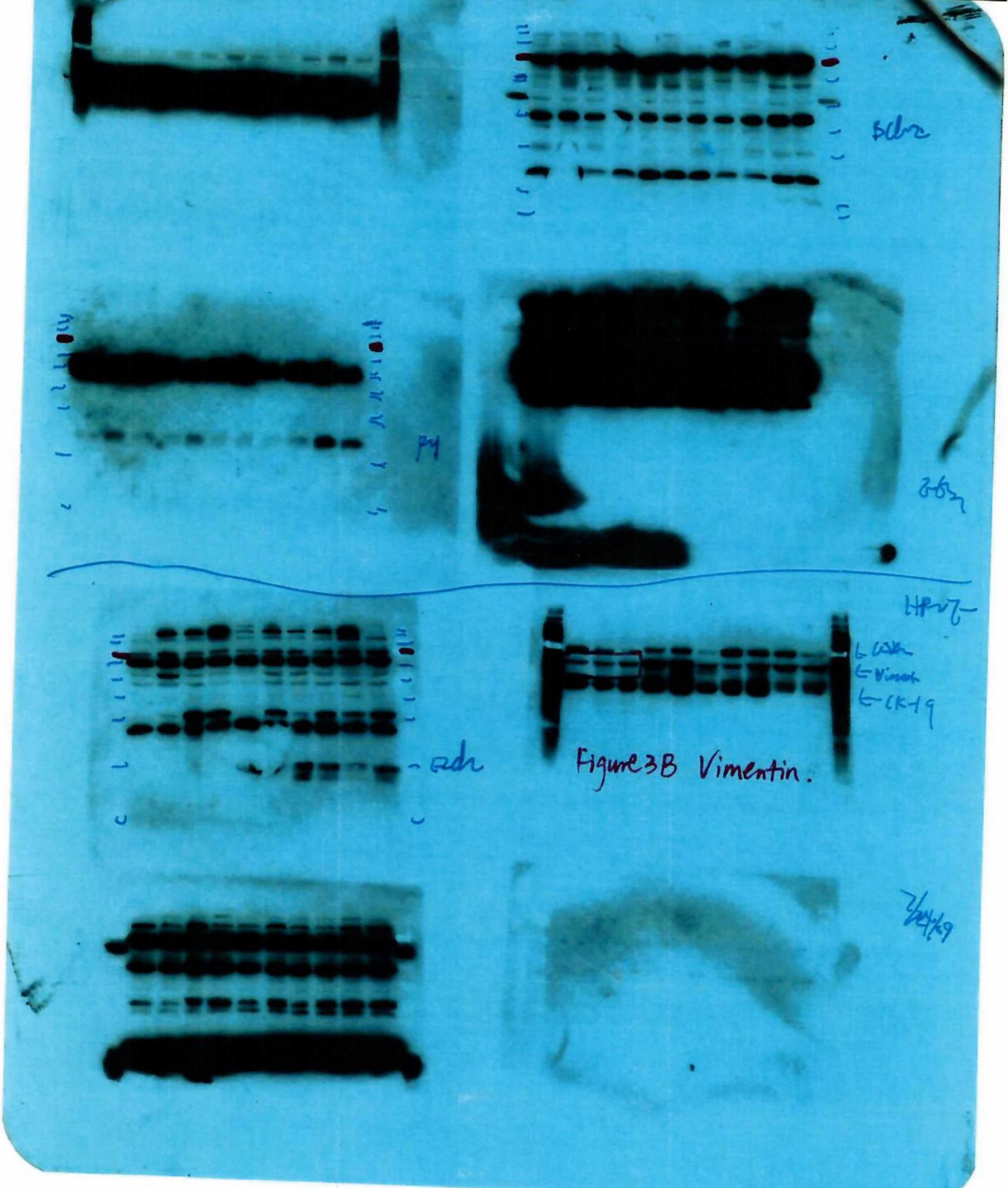
Figure3B Vimentin.

P8

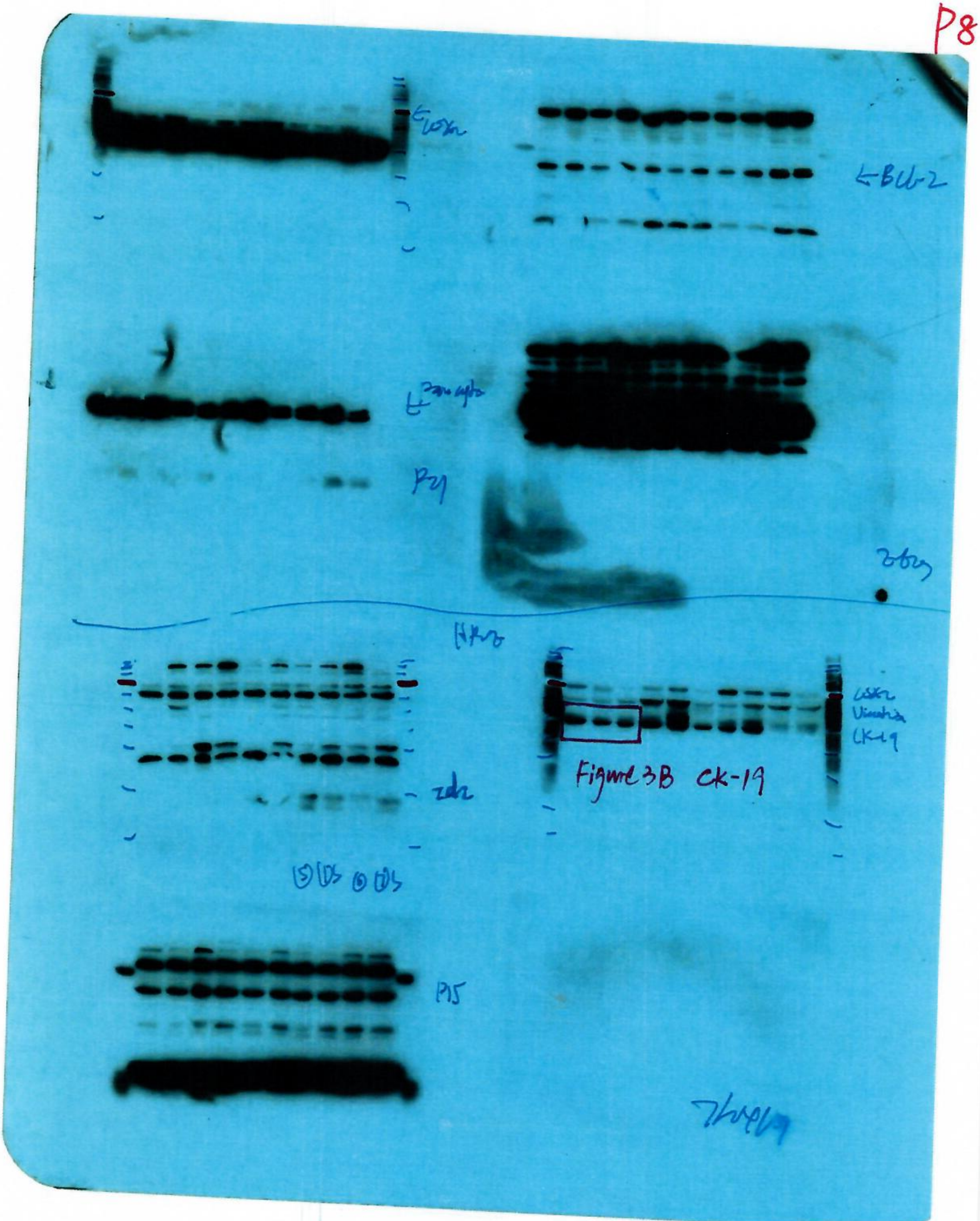

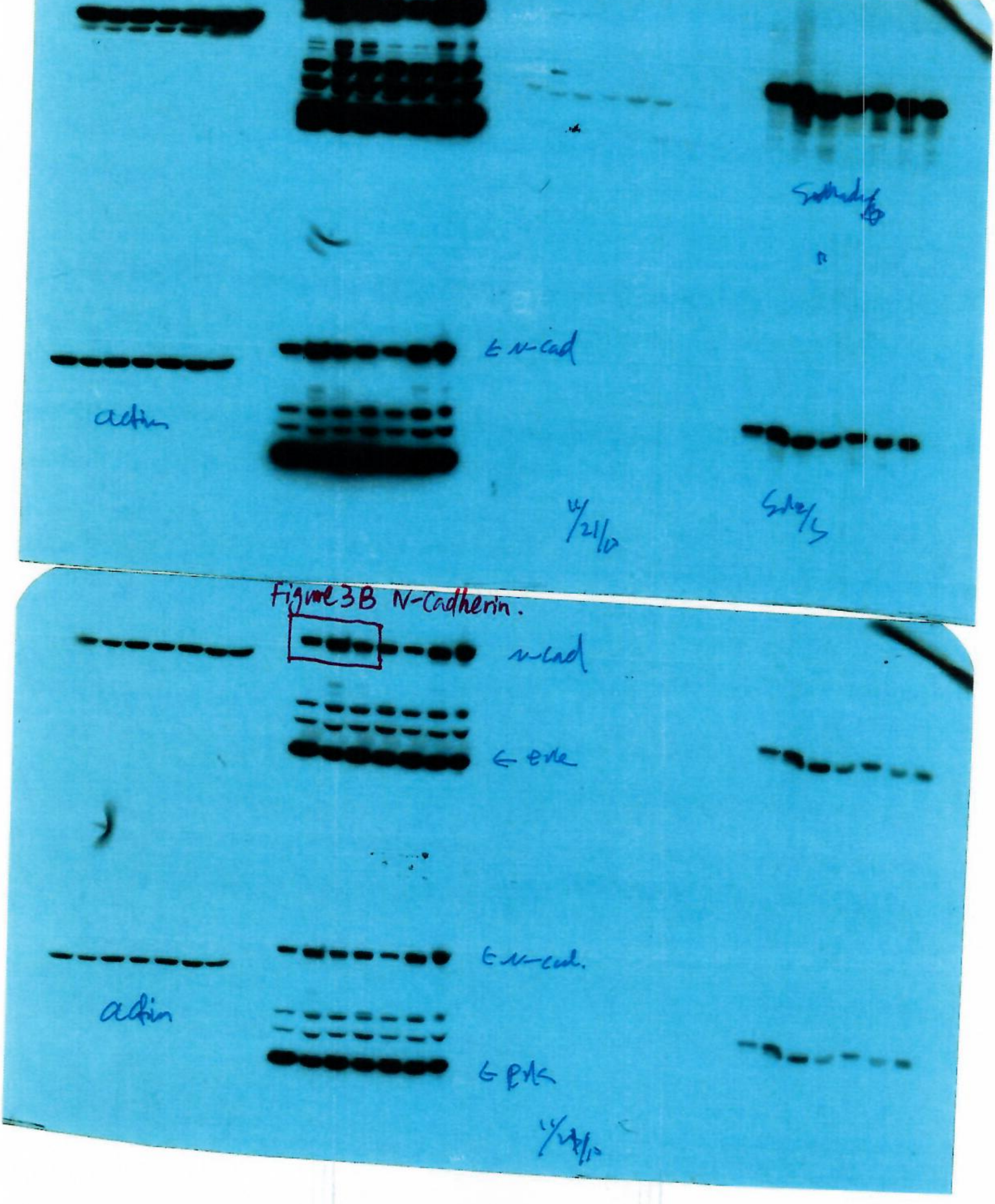
← N-cad
actin
Figure 3B N-Cadherin.
N-cad
← erk
← N-cad.
actin
← ERK

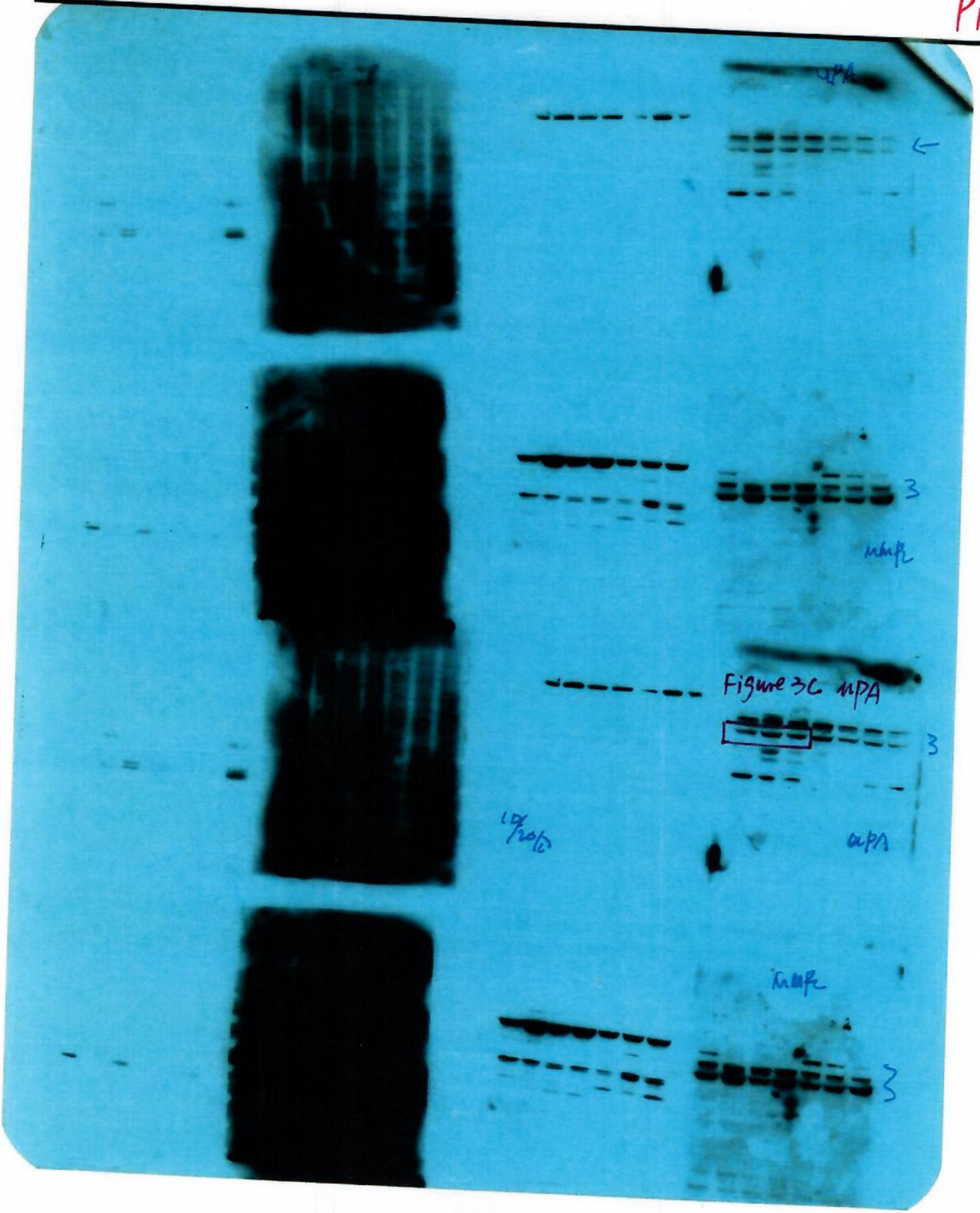
mmp2
Figure 3C uPA
uPA
mmp2

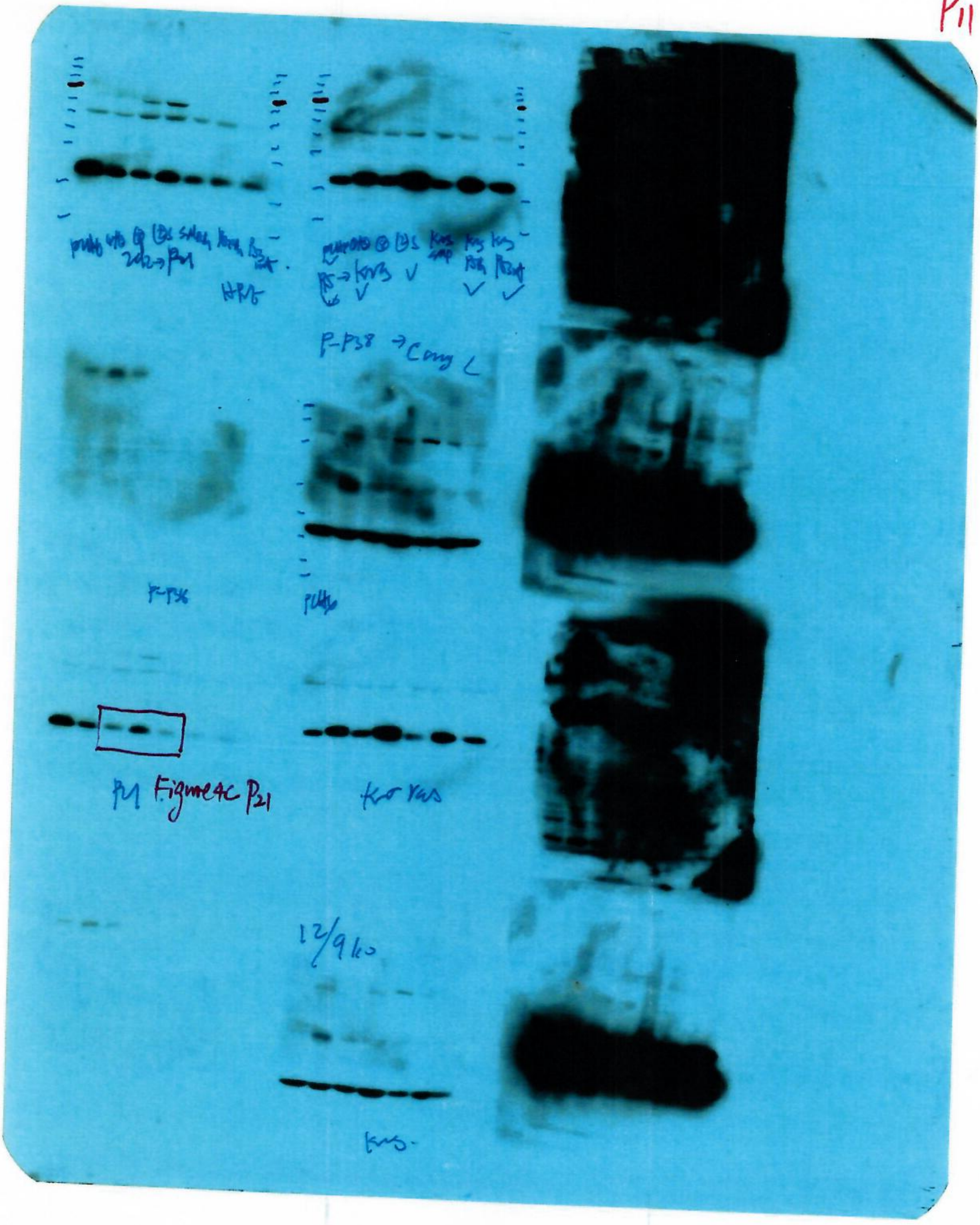
P-P38
Figure4C P21

P12

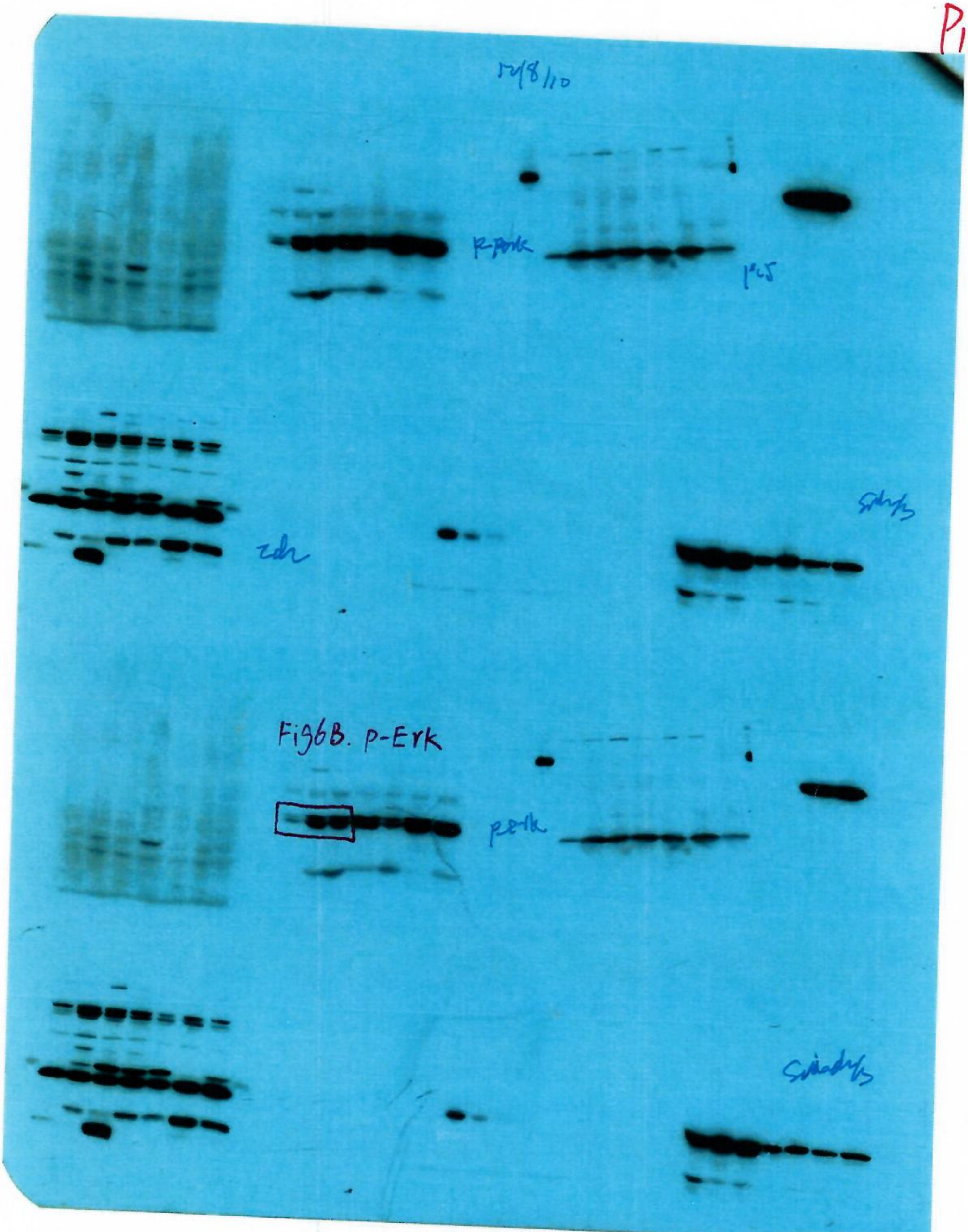

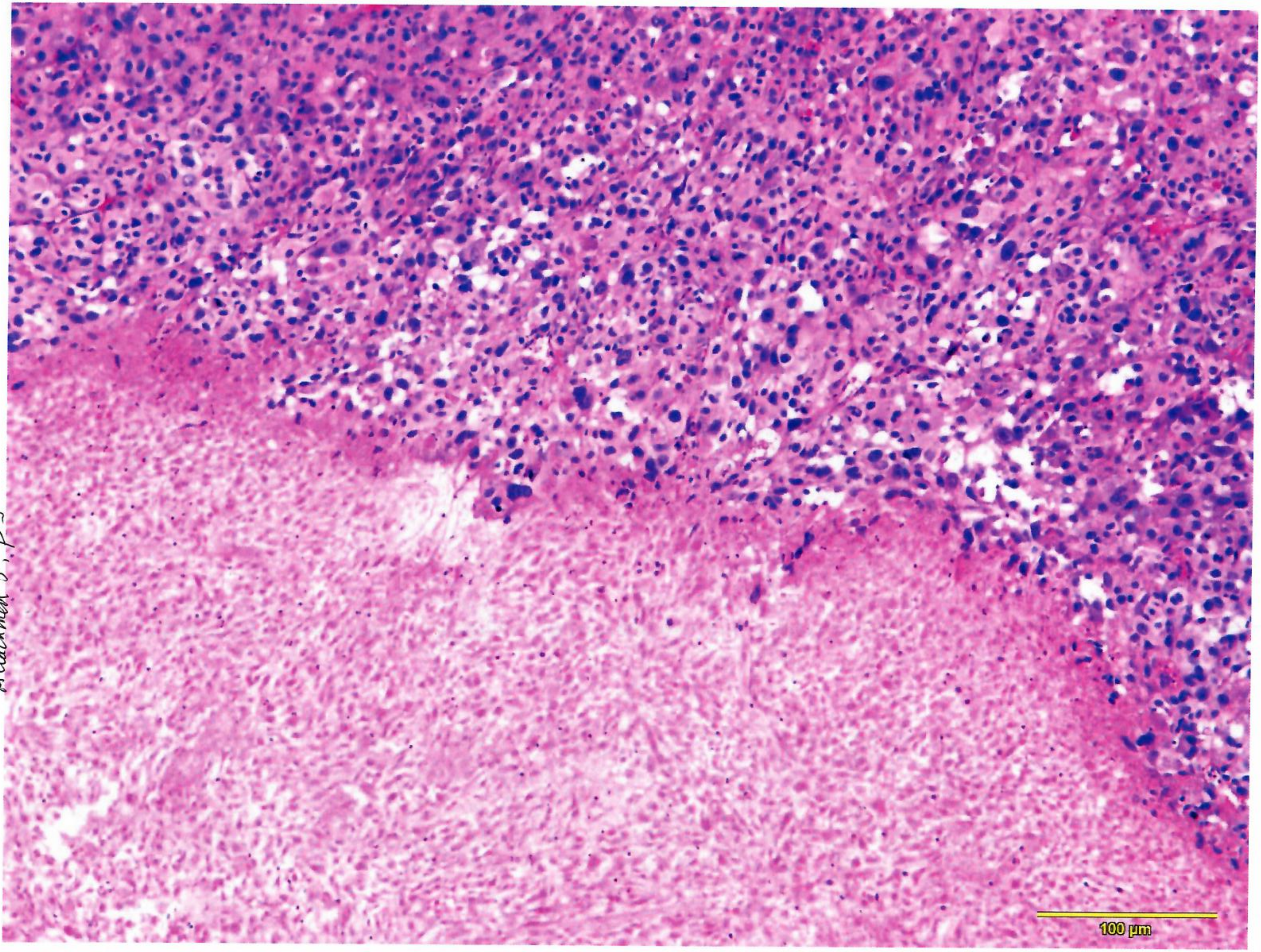

Attachment 5, p 5

Attachment 5 p6

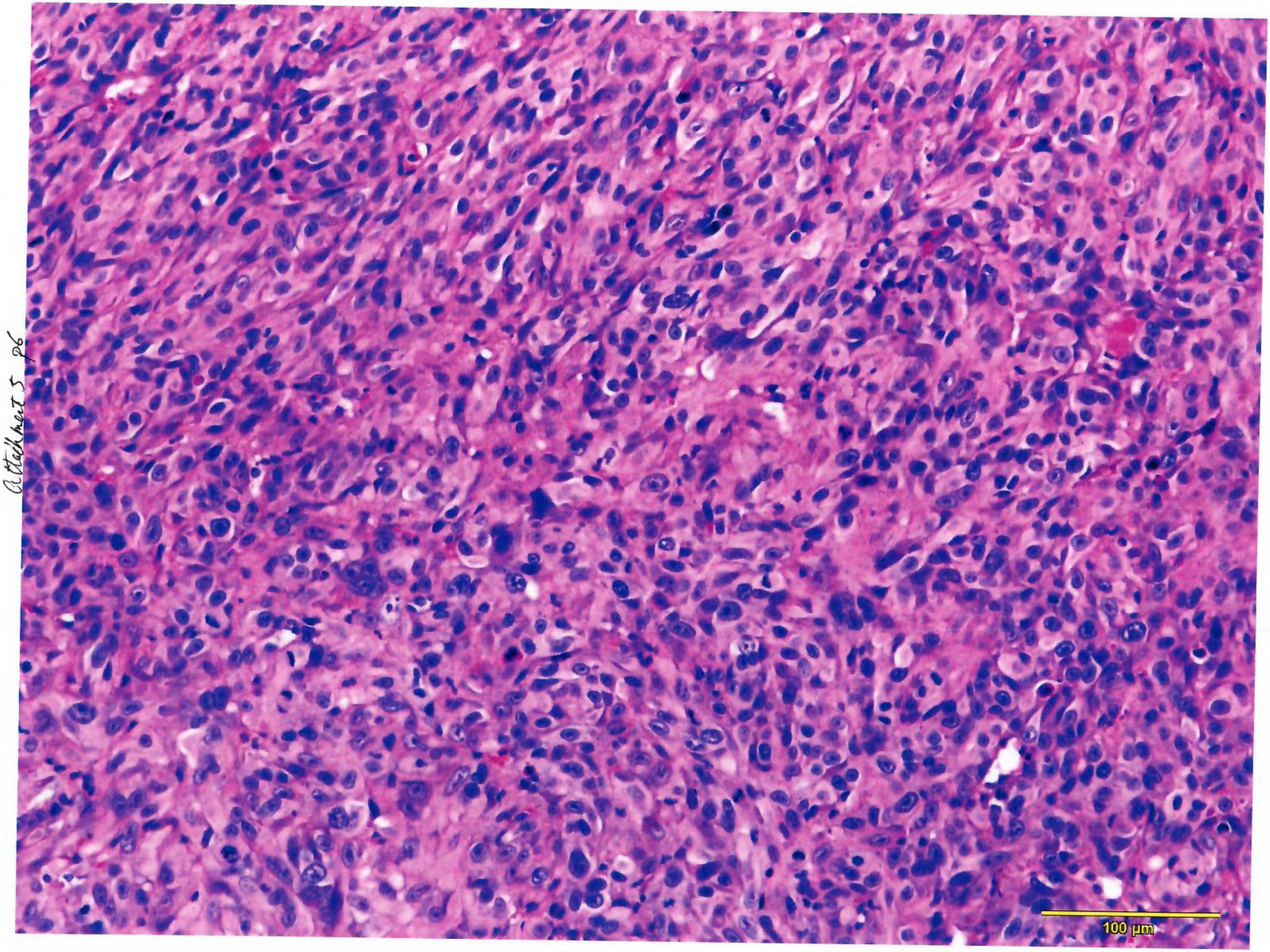

Attachment 5, p7

HER2

HPNE/K-ras/p16sh | Human Pancreatic cancer | Normal Pancreatic Duct

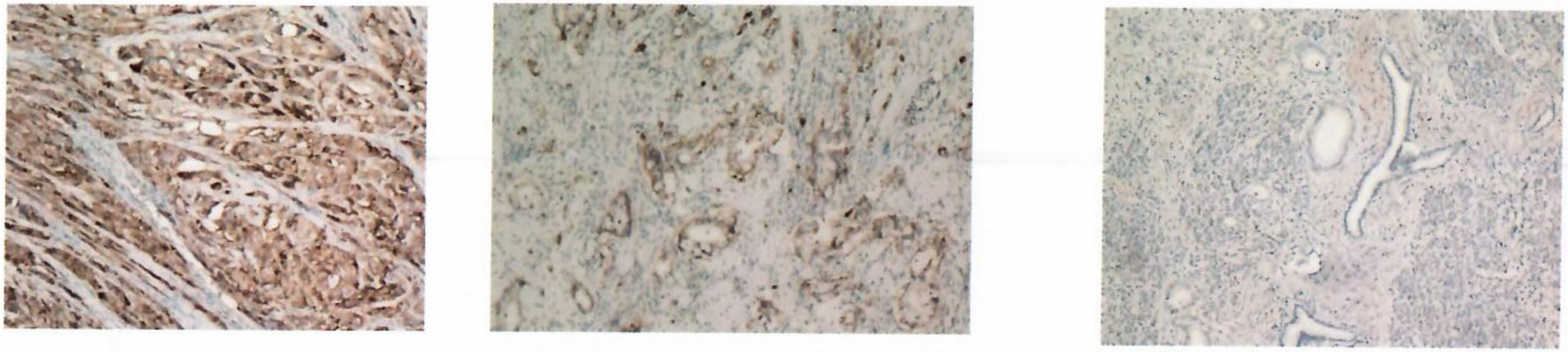

EGFR

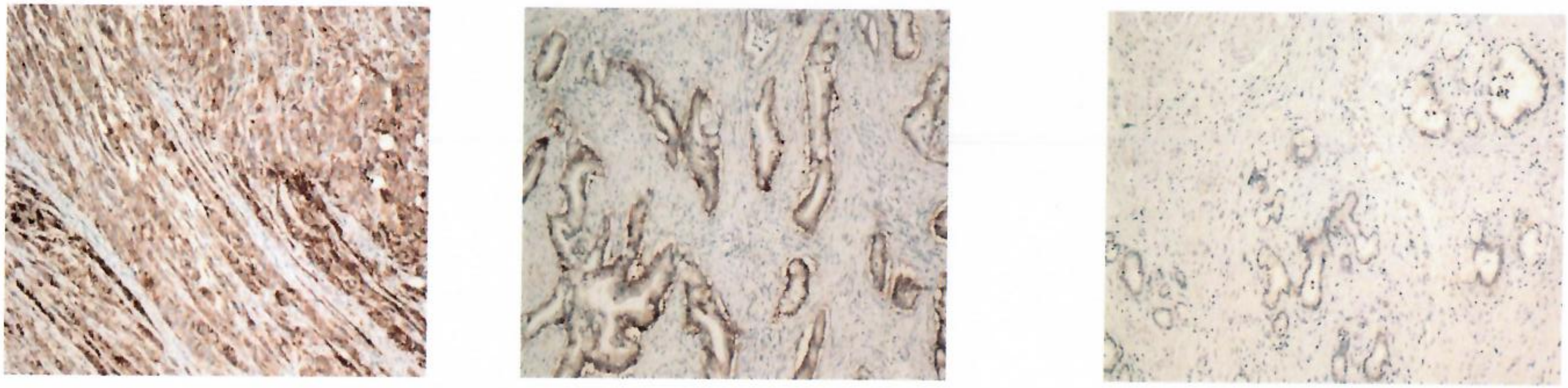

Attachment I, P2

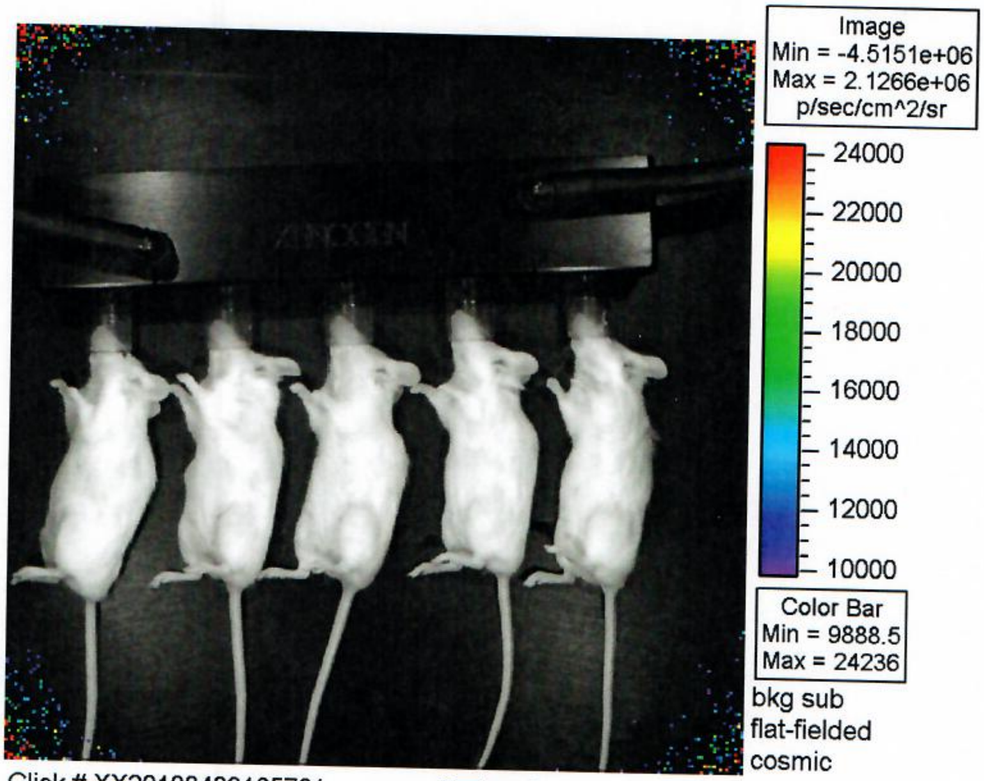

Click # XX20100409165721
Fri, Apr 09, 2010 16:58:53
Bin:M (8), FOV25, f1, 1m
Filter: Open
Camera: IVIS 13112, SI620EEV

Series: 2
Experiment:
Label: 111709
Comment: es
Analysis Comment:

Image
Min = -1.4178e+06
Max = 2.4816e+06
p/sec/cm^2/sr

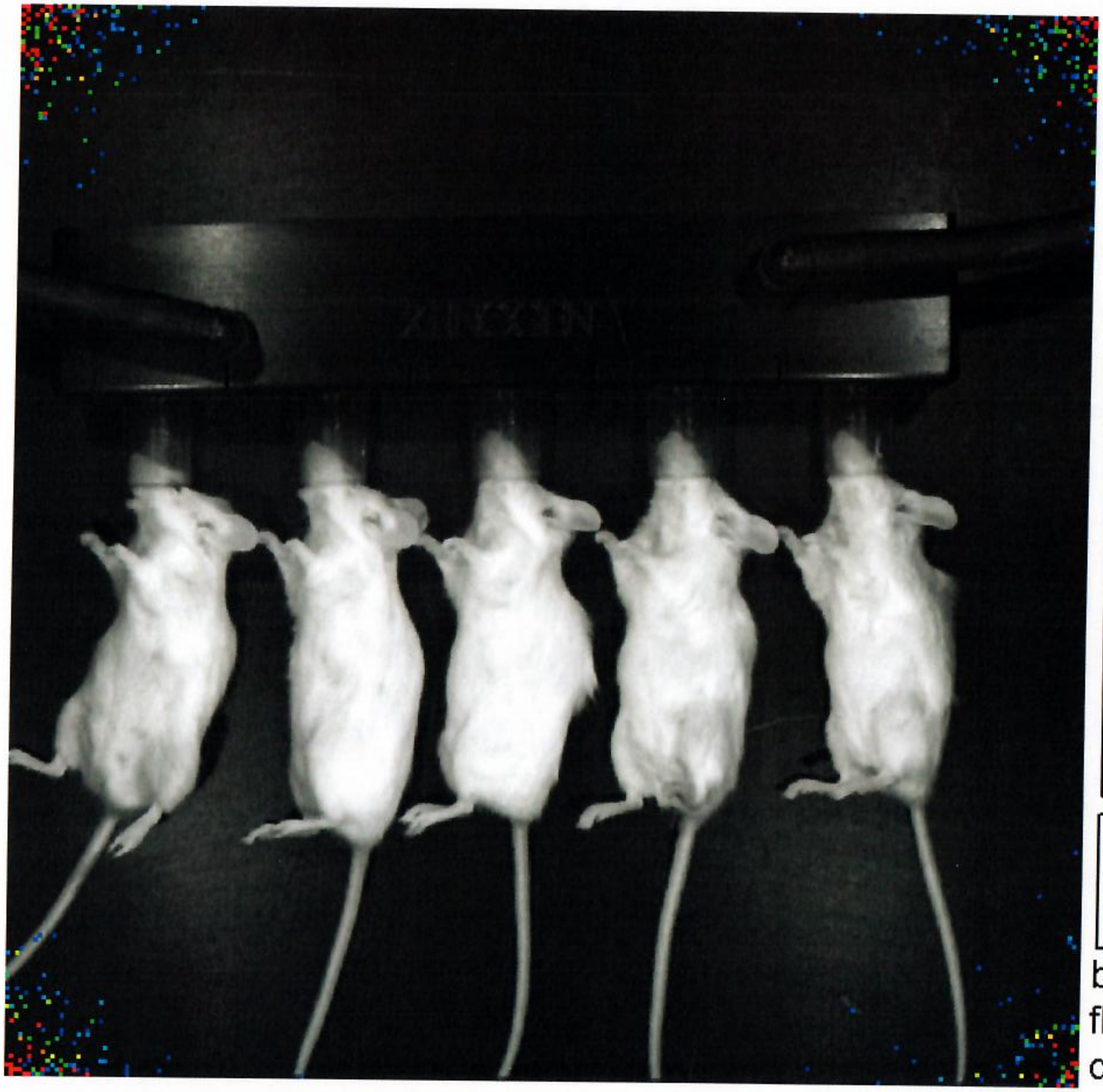

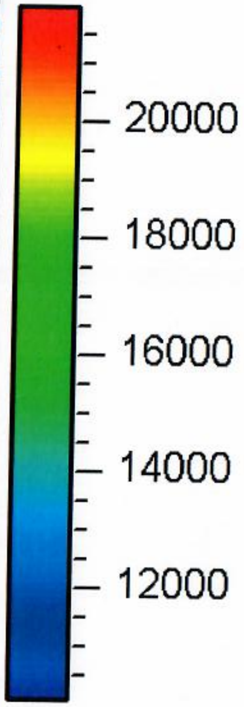

Color Bar
Min = 10157
Max = 21899

bkg sub
flat-fielded
cosmic

Click # XX20100409164028
Fri, Apr 09, 2010 16:40:53
Bin:M (8), FOV25, f1, 1m
Filter: Open
Camera: IVIS 13112, SI620EEV

Series: 2
Experiment:
Label: 111709
Comment: 9
Analysis Comment:

Attachment 5, p3

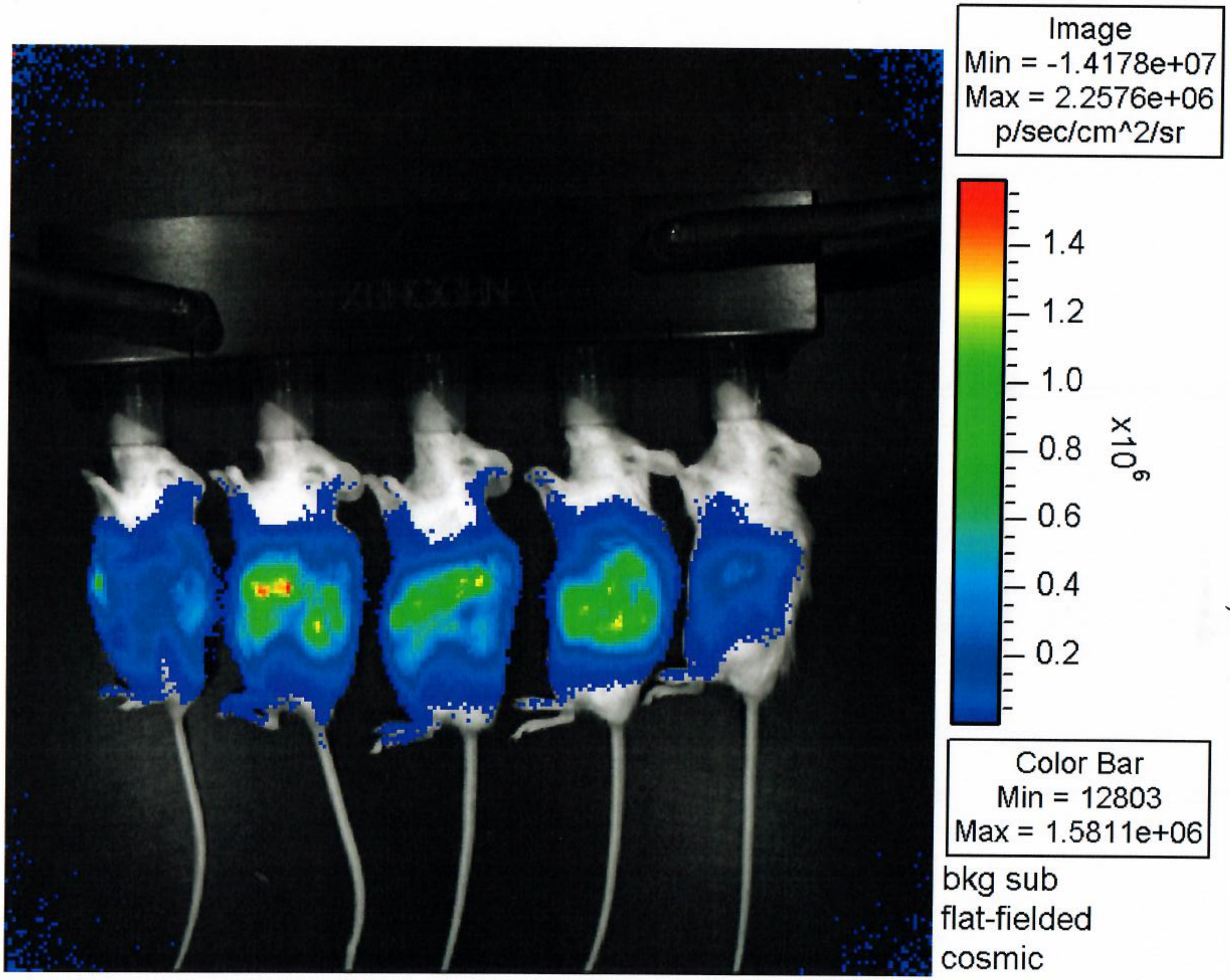

Attachment 5, P4

Click # XX20100409162614
Fri, Apr 09, 2010 16:26:32
Bin:M (8), FOV25, f1, 1m
Filter: Open
Camera: IVIS 13112, SI620EEV

Series: 2
Experiment:
Label: 111709
Comment: 8
Analysis Comment:

Attachment 3

HPNE/Vect	HPNE/Kras	HPNE/Kras/p16	HPNE/Kras/p16	HPNE/Kras	HPNE/Kras	HPNE/Kras	HPNE/Kras	HPNE/Kras	HPNE/Kras/Her2/p16p14shRNA/Smad4shRNA
2	99	146	142	84	74	125	87	92	69
3	96	160	126	78	90	100	86	102	65
2	98	156	136	82	83	108	84	93	59
2	102	140	150						
HPNE/Vect	HPNE/Kras	HPNE/Kras/p16	HPNE/Kras/p16	HPNE/Kras	HPNE/Kras	HPNE/Kras	HPNE/Kras	HPNE/Kras	HPNE/Kras/Her2/p16p14shRNA/Smad4shRNA
2.333333	97.66667	154	134.6666667	81.33333	82.33333	111	85.66667	95.66667	64.33333
0.57735	1.527525	7.211102551	8.082903769	3.05505	8.020806	12.76715	1.527525	5.507571	5.033223

3.68678E-06 0.001469222

2C

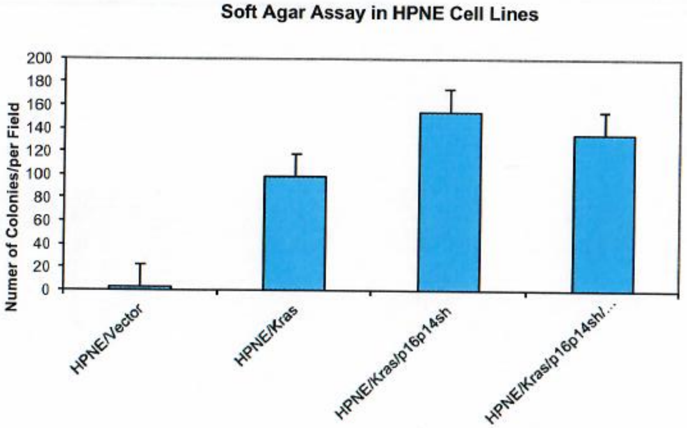

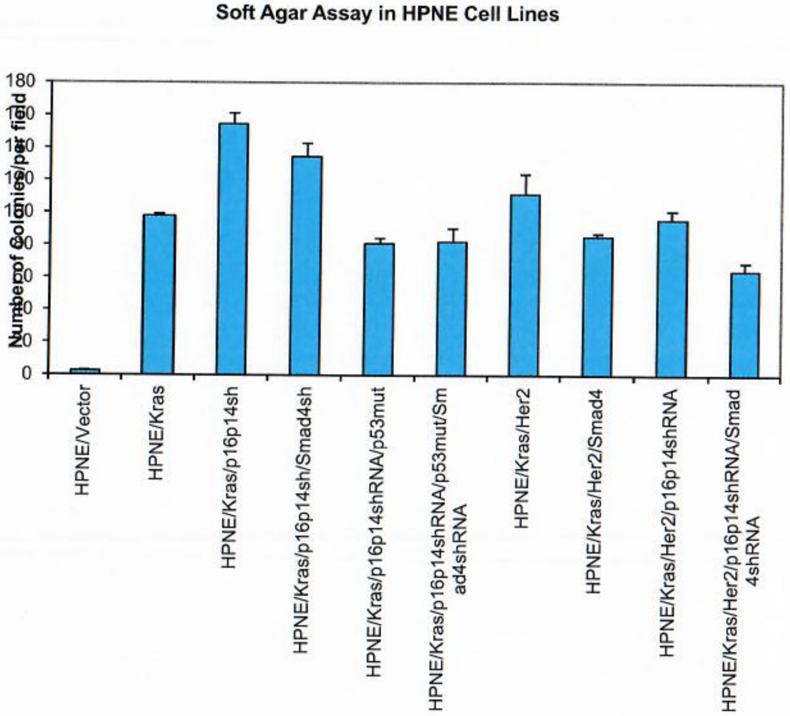

Attachment 4 *Fig4. B*

HPNE			HPNE/K-ras			HPNE/K-ras/p16sh	
40	3200	3.2	190	15200	15.2	13	1040
42	3360	3.36	180	14400	14.4	10	800
28	2240	2.24	176	14080	14.08	15	1200
23	1840	1.84	100	8000	8	12	960
20	1600	1.6	60	4800	4.8	13	1040
31	2480	2.48	42	3360	3.36	11	880
21	1680	1.68	50	4000	4	17	1360
45	3600	3.6	100	8000	8	11	880
		2.5			8.98		
		0.795128			4.92592		
					0.002457		

HPNE	HPNE/K-ra	HPNE/K-ras/p16sh
2.5	9.77	1.02
0.795128	5.589613	0.185164

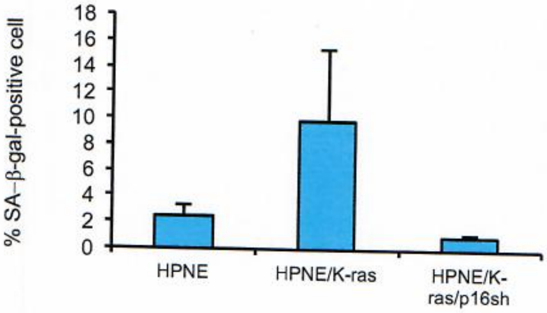

	HPNE	HPNE/K-ras	HPNE/K-ras/p16sh
1.04	0.032	0.152	0.0104
0.8	0.0336	0.144	0.008
1.2	0.0224	0.1408	0.012
0.96	0.0184	0.08	0.0096
1.04	0.016	0.048	0.0104
0.88	0.0248	0.0336	0.0088
1.36	0.0168	0.04	0.0136
0.88	0.036	0.08	0.0088
1.02		0.002457	0.001319
0.185164		0.00133	
0.001319			
